# A Sensor-Centric Survey of Autonomous Driving: Integrating Measurement Physics, Uncertainty Modeling, and Safety-Critical Multi-Sensor Fusion

**DOI:** 10.3390/s26123801

**Published:** 2026-06-15

**Authors:** Umar Iqbal, Ali Massoud, Aboelmagd Noureldin

**Affiliations:** 1 Department of Electrical Engineering, College of Engineering, Illinois State University, Campus Box 6000, Normal, IL 61790, USA; 2 Department of Electrical and Computer Engineering, Royal Military College of Canada, Kingston, ON K7K 7B4, Canada; ali.massoud@queensu.ca (A.M.); or nourelda@queensu.ca (A.N.); 3 Department of Electrical and Computer Engineering, Queen’s University, Kingston, ON K7L 3N6, Canada

**Keywords:** autonomous driving, sensor fusion, measurement physics, uncertainty quantification, integrity monitoring, SOTIF, ISO/PAS 8800, risk-aware planning, cooperative perception, safety assurance

## Abstract

Autonomous driving systems (ADSs) are reliable only when heterogeneous sensors, estimation algorithms, and safety mechanisms are engineered as a single coherent safety-critical measurement system rather than as loosely coupled modules. Production stacks integrate cameras, LiDAR, automotive radar, and GNSS/IMU, yet deployment remains constrained by modality-specific failure modes, calibration and synchronization drift, and out-of-distribution (OOD) conditions that violate modeling assumptions. These limitations induce overconfidence and downstream decision errors whenever planning assumes certainty sharper than sensing can justify. This survey introduces a sensor-centric framework linking measurement physics, uncertainty propagation, fusion integrity, safety assurance, and risk-aware planning and control. We formalize what each modality physically measures; unify probabilistic, evidential, and conformal uncertainty representations; analyze filtering, factor-graph, BEV, transformer, and state-space fusion architectures with an emphasis on robustness and graceful degradation; and generalize aviation-style integrity concepts (RAIM/ARAIM) to multi-modal autonomy. The distinctive contribution is a single sensor-to-assurance throughline in which every uncertainty representation is tied to its measurement physics, every fusion architecture is evaluated against an explicit integrity-monitoring requirement generalized from RAIM/ARAIM, and every safety-standard clause is mapped to a concrete architectural mechanism. We map these mechanisms onto ISO 26262, ISO 21448 (SOTIF), ISO/PAS 8800, ANSI/UL 4600, and the UNECE framework, and connect perception uncertainty to decision-making through chance-constrained MPC and formal safety filters (RSS, CBF). Industry case studies and emerging V2X and generative-simulation approaches close the loop to deployable safety arguments.

## 1. Introduction

Autonomous driving has advanced rapidly owing to breakthroughs in deep learning, sensor hardware, and embedded computing, motivating a substantial body of literature on learning-based perception, planning, and validation [1,2,3,4,5]. Despite this progress, a systemic gap persists: existing surveys address these challenges in isolation, focusing on either AI perception benchmarks, individual sensor modalities, or regulatory processes. These fragmented treatments lack a cohesive measurement-to-assurance framework that explains how failures originate at the hardware level, propagate through neural networks and estimation pipelines, and ultimately manifest as system-level safety risks [1,2,3,6]. Recent contributions have begun to bridge this gap by connecting uncertainty methods to perception [6], formalizing Safety of the Intended Functionality (SOTIF) as an emerging challenge [7], and surveying AI safety assurance across research, standardization, and regulation [8]. Yet none provides an end-to-end synthesis that unifies measurement physics (Section 3), structured uncertainty representation (Section 4), multi-sensor fusion under robustness and integrity constraints (Section 6), and system-level safety standards (Section 7) within a single coherent framework.

A sensor-centric stance begins with a foundational observation: every downstream autonomy decision is conditioned on a finite set of physical measurements with non-ideal error structures. Cameras measure scene irradiance projected onto pixel arrays, yielding rich semantics but remaining fundamentally ambiguous with respect to metric depth [5,9]. LiDAR measures optical time-of-flight or phase-based laser returns, providing precise metric geometry but suffering atmospheric attenuation and backscatter during precipitation [10,11]. Automotive radar measures radio-frequency beat frequencies and Doppler shifts, offering robust performance in reduced-visibility conditions but constrained by angular resolution and multipath effects [12,13]. GNSS receivers and IMUs provide absolute timing, positioning, and inertial continuity, yet remain vulnerable to multipath distortion in urban canyons and to bias-driven drift [14,15]. Crucially, the autonomy variables necessary for safe navigation, 3D object bounding boxes, drivable free space, pedestrian intent, and collision-risk probabilities are largely inferred rather than directly observed. Inference fragility, therefore, becomes the dominant failure mode under distribution shift and adverse conditions, a challenge that nominal accuracy on curated benchmarks consistently underestimates [16,17].

The central technical challenge extends well beyond improving nominal detection accuracy on static datasets. It demands architectures that (i) represent uncertainty realistically, capturing both irreducible sensing noise and model-driven epistemic uncertainty; (ii) fuse heterogeneous measurements into consistent, calibrated state and scene estimates; (iii) monitor system integrity to detect assumption violations, calibration drift, and out-of-distribution (OOD) exposure; and (iv) enforce safe fallback behaviors when confidence degrades below operational thresholds [18,19,20]. This perspective aligns directly with the regulatory imperative to eliminate unreasonable risk: functional safety standards address deterministic hardware malfunctions [21]; SOTIF additionally targets hazards from performance insufficiencies even in the absence of component faults [22]; ISO/PAS 8800 extends both frameworks to the non-deterministic risks of ML-based automotive systems [23]; and UL 4600 frames ADS safety as an evidence-based argument for systems operating without human supervision [24]. Together, these standards demand traceable assurance arguments supported by integrity monitoring, risk-aware operational governance, and rigorous scenario-based validation [25,26].

Contributions. The distinctive contribution of this survey is a single sensor-to-assurance throughline in which (a) every uncertainty representation is tied to the measurement physics that produces it, (b) every fusion architecture is evaluated against an explicit integrity-monitoring requirement generalized from RAIM/ARAIM, and (c) every safety-standard clause, ISO 26262 [21], ISO 21448 [22], ISO/PAS 8800 [23], UL 4600 [24], and the UNECE GTR on ADSs [26], is mapped to a concrete architectural mechanism. This combination of commitments distinguishes the present survey from prior work in the area. This survey makes four contributions. First, it establishes a direct connection between modality-level measurement physics, what each sensor directly observes, which task variables must be inferred, and why observability limits define characteristic failure modes, and structured uncertainty and error propagation models (Section 3 and Section 4). Second, it treats calibration and temporal synchronization as first-order determinants of fusion quality rather than implementation details (Section 4). Third, it synthesizes multi-sensor fusion architectures spanning Kalman-family filters, factor graphs, BEV deep fusion, transformer cross-modal attention, state-space models (SSMs), and resilient expert-routing architectures through the integrated lenses of uncertainty realism, integrity monitoring, and real-time deployability (Section 6). Fourth, it connects these technical mechanisms to functional safety (ISO 26262), SOTIF (ISO 21448), AI safety (ISO/PAS 8800), safety-case practice (ANSI/UL 4600), and regulatory requirements, including the UNECE draft Global Technical Regulation (GTR) on ADSs, adopted by GRVA on 23 January 2026 and pending formal WP.29 adoption (anticipated June 2026), and to risk-aware planning and control (Section 7, Section 8, Section 9 and Section 10) [21,22,23,24,25,26].

Differentiation from prior surveys. Existing surveys cover overlapping but disjoint slices of the problem. Surveys of self-driving and deep-learning-based perception [1,2,3,4,5] catalog algorithms and benchmarks but treat sensors as fixed inputs and do not formalize how measurement physics constrains downstream safety. Multi-modal perception and sensor-fusion reviews [4,27] map fusion architectures yet stop short of integrating uncertainty propagation, integrity monitoring, and standards compliance. Uncertainty-focused reviews [6,28,29] consolidate probabilistic and evidential methods but do not connect them to fusion robustness, ODD-aware fallback, or auditable safety cases. SOTIF surveys [7] articulate the performance-insufficiency challenge but remain abstract regarding the sensor-level mechanisms that satisfy it. AI-safety standardization surveys [8,30] examine regulatory and verification practices without grounding in sensor measurement principles. The present survey’s distinctive contribution is to weave these strands into a single sensor-to-assurance throughline: every claim about uncertainty representation, fusion architecture, or planning margin is traced back to a specific measurement principle, and every safety-standard requirement is mapped to a concrete architectural mechanism. To our knowledge, no prior survey unifies measurement physics, calibrated uncertainty propagation, fusion integrity, and the ISO 26262/ISO 21448/ISO/PAS 8800/UL 4600/UNECE compliance landscape under a common framework.

**Figure 1:**
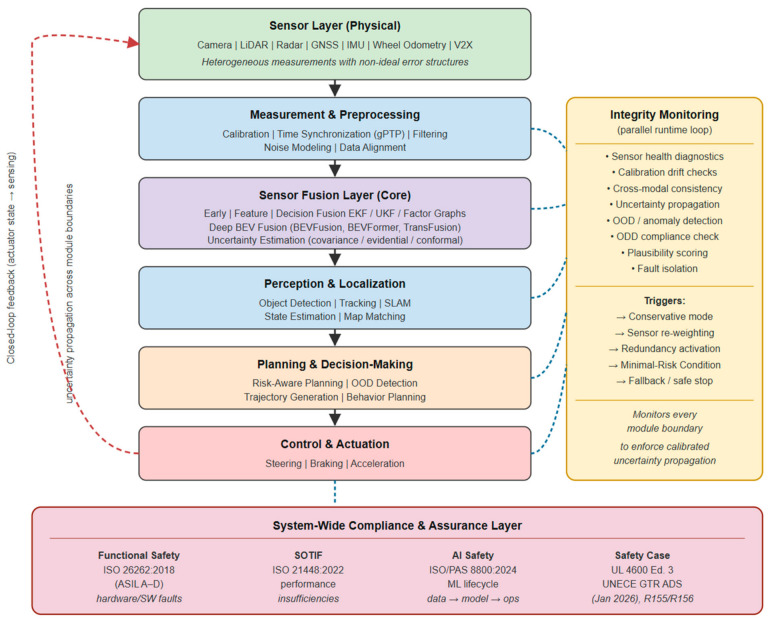
Sensor-to-assurance pipeline for autonomous driving. Raw observations from cameras, LiDAR, radar, GNSS, IMU, wheel odometry, and V2X are refined through measurement and preprocessing (calibration, time synchronization via gPTP, filtering), fused at the sensor-fusion layer (filtering, factor graphs, BEV deep fusion), and consumed by perception, planning, and control modules; a parallel integrity-monitoring loop evaluates sensor health, calibration drift, cross-modal consistency, out-of-distribution (OOD) exposure, and operational design domain (ODD) compliance, triggering conservative mode, redundancy activation, or minimal-risk conditions (MRCs) when thresholds are crossed. A system-wide compliance and assurance layer aligns the stack with ISO 26262, ISO 21448 (SOTIF), ISO/PAS 8800, ANSI/UL 4600, and the UNECE GTR on ADSs. Box colors distinguish the architectural layers (green: physical sensor layer; blue: measurement/preprocessing and perception; purple: sensor fusion; orange: planning; red: control; yellow: integrity monitoring; dark red: compliance and assurance); solid black arrows denote the forward data flow, blue dotted connectors link each module boundary to the integrity monitor, and the red dashed arrow denotes the closed-loop feedback from actuation back to sensing.

The remainder of this paper is organized as follows. Section 2 provides a system-level overview of ADS architectures and the role of uncertainty propagation across module boundaries. Section 3 establishes sensor fundamentals. Section 4 formalizes error sources, calibration, and uncertainty representations. Section 5 analyzes environmental and scenario challenges. Section 6 surveys multi-sensor fusion architectures. Section 7 maps sensing and fusion mechanisms to safety standards. Section 8 analyzes redundancy and design trade-offs. Section 9 examines industry deployments. Section 10 connects perception uncertainty to risk-aware planning and control. Section 11 synthesizes research directions. Section 12 concludes. Figure 1 illustrates the overall sensor-to-assurance pipeline that structures the survey.

## 2. Autonomous Driving System Overview

Autonomous driving systems are organized as layered, tightly coupled architectures that transform raw sensor measurements into safe control actions through a sequence of perception, localization, prediction, planning, and control modules. While this modular decomposition is widely adopted [1,2,3], the effectiveness of each module is fundamentally constrained by upstream sensing limitations and by the way uncertainty propagates across module boundaries. The sensor-centric perspective adopted here treats the entire stack as a single safety-critical measurement-and-assurance pipeline in which each module’s outputs must carry calibrated confidence information forward to downstream consumers.

### 2.1. Core System Modules

The perception layer ingests raw sensor streams and produces semantic and geometric outputs, object detections, tracks, occupancy grids, lane markings, traffic signs, and free-space boundaries, typically via BEV-based neural architectures such as BEVFusion [31] and BEVFormer [32]. The localization layer estimates ego pose by fusing GNSS pseudorange observables, IMU integration, wheel odometry, and map-matching residuals through EKF, UKF, or factor-graph backends [14,33,34]. The prediction layer forecasts other agents’ trajectories [35,36] and must propagate upstream perception uncertainty into multi-modal behavior distributions. The planning layer converts perception and prediction into kinematic trajectories under dynamics, actuator, and safety constraints [37], and the control layer translates desired trajectories into steering, throttle, and braking commands. A compliance and safety layer, integrating ISO 26262 [21], SOTIF [22], ISO/PAS 8800 [23], UL 4600 [24], and evolving UNECE regulations [26], spans all modules and enforces runtime safety policies.

### 2.2. Sensor Data Flow and Uncertainty Propagation

A central principle of this survey is that autonomy safety depends on propagated uncertainty rather than on local confidence scores at individual module outputs. Object-detection confidences must propagate into track-manager covariances, then into prediction distributions, and then into planner collision-risk estimates and safety margins; localization covariances must propagate into map-relative reasoning and lane-boundary precision. Without consistent propagation, systems accumulate overconfidence as each module discards uncertainty information, and the planner operates on point estimates that have lost their provenance [6,16,18]. Modern architectures, therefore, treat uncertainty as a first-class signal through explicit covariance tracking in classical filters, aleatoric/epistemic heads in learned perception modules [18], evidential outputs [38,39], or conformal prediction sets [20] and propagate it through the entire pipeline.

From sensing failure to system-level safety risk. The relevance of uncertainty propagation is best appreciated by tracing how a single sensor-level deficiency cascades through the pipeline into a hazardous control action. Consider a 1° extrinsic-rotation drift between a forward camera and the LiDAR, an undetected calibration fault that produces a 1.75 m lateral displacement at 100 m range (Section 4.2). At the perception layer, this manifests as cross-modal misassociation: a vulnerable road user detected by the camera is fused with the wrong LiDAR point cloud, resulting in a track whose position covariance underestimates the true error. The tracker, receiving an apparently consistent measurement, reports tight covariance to the predictor, which then forecasts trajectories with falsely narrow uncertainty bounds. The planner’s chance-constrained collision check (Section 10.2) consumes this overconfident input and accepts a gap-acceptance maneuver whose true collision probability exceeds the safety budget. A symmetric cascade can be initiated by GNSS multipath in an urban canyon, LiDAR backscatter in fog, IMU bias drift in a long tunnel, or an OOD object class encountered for the first time. In each case, the root cause is a sensor-level departure from modeling assumptions, but the harm surfaces only at the actuation boundary, and only because the intervening modules failed to honestly propagate the sensor’s degraded confidence. This causal pattern motivates the sensor-centric stance of this survey. Every safety claim about the planner or controller is conditional on a faithful uncertainty record that begins at measurement acquisition and is preserved across every module boundary.

### 2.3. On-Board, Edge, and Cloud Computation

Production ADS partition compute between on-board perception/control running on automotive SoCs and off-board functions such as HD map updates, fleet learning, and long-term storage. On-board compute dominates safety-critical real-time paths; edge and cloud computation complement it with higher-latency services but introduce connectivity dependencies and potential attack surfaces [40]. Mixed-criticality architectures typically isolate the integrity monitor and minimal-risk-condition (MRC) controller on a lockstep microcontroller separate from the high-throughput AI accelerator, so that fallback logic continues to execute even if the primary perception pipeline stalls or violates timing assertions.

### 2.4. V2X Communication and Cooperative Perception

Vehicle-to-everything (V2X) communication extends onboard observability beyond line-of-sight by enabling the exchange of basic safety messages, collective perception messages, and cooperative maneuver data among vehicles and roadside units [41,42,43,44]. Architectures such as V2X-ViT [41] and Where2comm [42] demonstrate cooperative perception pipelines that aggregate features across multiple agents while managing bandwidth constraints. From a safety perspective, cooperative perception must be integrated through trust-aware fusion: cryptographically authenticated messages do not guarantee veracity, since a compromised or degraded sender may broadcast plausible but incorrect state, necessitating plausibility scoring against onboard evidence (Section 11.2).

### 2.5. System-Level Safety Integration

**Figure 2:**
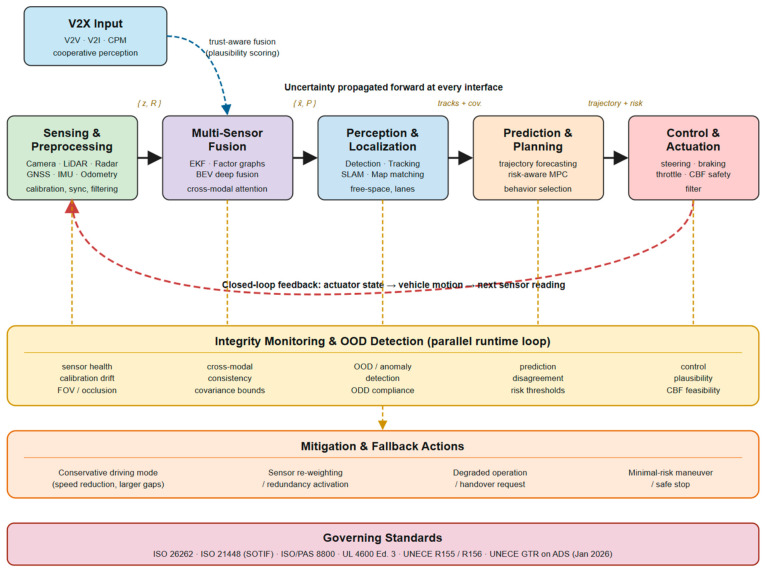
Closed-loop ADS architecture emphasizing uncertainty propagation across module boundaries. Onboard sensing and V2X inputs flow through multi-sensor fusion, perception/localization, prediction/planning, and control/actuation, with every interface carrying calibrated confidence information forward. Integrity monitoring runs in parallel, detecting sensor faults, OOD events, prediction disagreement, and control plausibility issues; mitigations range from conservative driving to sensor re-weighting, degraded operation, or a minimal-risk maneuver. Governance is supplied by ISO 26262, ISO 21448 (SOTIF), ISO/PAS 8800, UL 4600 Ed. 3, and UNECE R155/R156 plus the January 2026 draft GTR on ADSs. Solid black arrows denote the forward data flow between modules; the blue dashed arrow denotes trust-aware injection of cooperative V2X inputs; the red dashed arrow denotes the closed-loop feedback from actuator state to the next sensor reading; and yellow dashed connectors link each interface to the parallel integrity-monitoring band, whose detections feed the mitigation layer below.

From a sensor-centric viewpoint, the autonomy stack is a closed-loop estimation-and-decision system in which sensing, fusion, planning, and control are continuously and bidirectionally coupled. Safety is not localized to any single module but emerges from the coordinated interaction of all components under uncertainty, degraded sensing, and ODD boundary conditions. As illustrated in Figure 2, the assurance layer’s mapping to specific standards (ISO 26262, ISO 21448, ISO/PAS 8800, UL 4600, and the UNECE regulatory family) is treated in detail in Section 7; here, we note only the architectural consequence: enforcement must occur across all layers and rests on three properties: (i) continuous integrity monitoring at each module boundary, (ii) redundancy and fault-tolerant design eliminating single-point safety failures, and (iii) risk-aware planning and fallback strategies that trigger minimum-risk conditions when confidence degrades below operational thresholds. Robust autonomy depends not only on accurate algorithms within individual modules but on consistent uncertainty management across the entire system.

## 3. Sensor Fundamentals and Measurement Principles

A key distinction in autonomous sensing is between directly measured quantities and inferred variables, where observability limitations fundamentally define achievable performance and failure modes. This section establishes a measurement-first view of the sensor stack: what each modality directly observes, what must be inferred, and how the physics of each measurement principle defines a characteristic error structure. Understanding these fundamentals is a prerequisite for the uncertainty modeling, fusion architecture, and safety-assurance discussions in Section 4, Section 5, Section 6 and Section 7, because every algorithm operating on sensor data inherits the limitations imposed at the point of physical acquisition.

### 3.1. Active vs. Passive Sensing

A foundational distinction in automotive sensing is between passive and active modalities. Passive sensors, primarily cameras, measure ambient scene radiance and produce high-resolution, semantically rich outputs, but cannot independently recover metric depth or velocity, since those quantities require knowledge of the energy source, its geometry, and propagation time. Active sensors, LiDAR, radar, and ultrasound, emit controlled energy and measure properties of the return (time-of-flight, frequency shift, power), enabling direct metric range and velocity observability. Passive sensors excel at classification and texture discrimination; active sensors anchor metric estimation and integrity monitoring [9,10,11,12]. Active sensing introduces eye-safety constraints on laser power budgets, waveform coexistence between vehicles in dense traffic, and susceptibility to atmospheric attenuation and mutual interference, engineering constraints that shape the operating envelope and must be treated as first-order uncertainty sources rather than edge cases [11,12,13].

### 3.2. Camera: Measurement Model and System Implications

A camera’s primary measurement is pixel irradiance, scene radiance integrated over the pixel aperture, modulated by lens transfer function, sensor quantum efficiency, and exposure duration [9,27]. Modern automotive imagers must satisfy two requirements historically in tension: high dynamic range (HDR) of order 120 dB and beyond, to handle simultaneous bright sky and deeply shadowed road surfaces; and LED flicker mitigation (LFM), to reliably detect pulse-width-modulated traffic signals and brake lights whose duty cycles can disappear from short-exposure HDR frames. The Sony IMX490, a 5.4 MP automotive imager qualified to AEC-Q100, achieves 120–140 dB HDR via Digital Overlap (DOL) exposure with built-in LFM [27], and OmniVision’s HALE (HDR and LFM Engine) targets the same trade-off with substantially lower power consumption [45]. LFM is, therefore, a measurement-integrity feature, not a cosmetic refinement: without it, a perception network can systematically miss illuminated traffic signals during their off-phase, resulting in a silent failure mode that is invisible to nominal-condition benchmarks. Rolling-shutter artifacts, spatial distortions introduced by line-by-line exposure during motion, are mitigated either by global-shutter architectures (which sacrifice low-light sensitivity) or by algorithmic motion compensation.

The fundamental observability limitation of a monocular camera is metric depth: without stereo baselines or learned priors, depth must be inferred from monocular cues such as object size, foreshortening, and texture gradients. Recent foundation-scale monocular depth estimators, Depth Anything V2 [46], Metric3D v2 [47], and Depth Pro [48], achieve metric depth recovery that is competitive with active sensors under nominal conditions but remain susceptible to distribution shifts and OOD illumination. Scale ambiguity and the absence of direct velocity observability motivate camera-plus-active-sensor fusion as the dominant automotive paradigm. The system-level implication is that depth and velocity errors from monocular inference are *silent* in the absence of ground-truth supervision: the network may produce high-confidence but geometrically incorrect outputs under OOD conditions, creating a failure mode that nominal benchmarks systematically underestimate [16,17]. Explicit epistemic uncertainty quantification (Section 4) is, therefore, a first-order requirement.

### 3.3. LiDAR: Ranging Physics and System Implications

Automotive LiDAR measures range through either time-of-flight (ToF) or frequency-modulated continuous-wave (FMCW) processing. In ToF systems, a laser pulse is emitted, and the round-trip time τ is measured, yielding the range R = cτ/2, where the pulse width and the receiver bandwidth determine the resolution. FMCW LiDAR transmits a chirped waveform and measures the beat frequency between transmitted and received signals, providing direct radial velocity measurements under coherent detection assumptions, a capability unavailable to ToF systems without multi-pulse processing [10,49]. The FMCW architectural advantage, simultaneous range and velocity, immunity to solar background, and rejection of mutual interference from other LiDARs come at the cost of long-coherence lasers and silicon-photonics integration that remain more expensive than ToF alternatives [10]. A representative 2025 production-grade FMCW system, Aeva’s Atlas Ultra [49], reportedly achieves a 250 m detection range for low-reflectivity targets (500 m maximum) with a 150° horizontal field of view at 1550 nm, and is reportedly deployed across Aeva’s Atlas-line FMCW LiDARs in production engagements with Daimler Truck (via Torc Robotics for the Freightliner Cascadia) and ZF. The broader implication is that FMCW promotes the transition from an inferred quantity to a directly observed per-point measurement, qualitatively changing what downstream tracking and prediction modules can verify.

Wavelength selection, predominantly 905 nm versus 1550 nm, defines a system-level trade among eye-safety constraints, detector cost, and atmospheric propagation. At 1550 nm, the cornea absorbs the laser before it reaches the retina, so the IEC 60825-1 Class 1 eye-safety budget permits substantially more transmitted optical power than at 905 nm; this translates into longer practical detection ranges and higher SNR at long range, but requires InGaAs photodetectors that are markedly more expensive than mature silicon APDs [10]. Cost asymmetry is decisive in practice: by available counts, the vast majority of deployed automotive LiDARs use a 905 nm wavelength [10]. Water absorption at 1550 nm is somewhat higher, partially offsetting the 1550 nm power advantage in heavy rain. The dominant environmental failure mode for both wavelengths is atmospheric attenuation and backscatter following the Beer–Lambert law P ∝ exp(−2ζR); controlled experiments quantify point-cloud degradation as a function of rain rate and meteorological optical range, with detection probability for distant objects falling well below 50% under heavy rain and dense fog [11,50,51,52,53,54]. Window contamination from droplets, ice, and mud produces near-field false returns that degrade both detection geometry and calibration-based extrinsic estimates. These degradation characteristics are strongly range-dependent, producing a heteroscedastic uncertainty structure that homoscedastic Gaussian noise models cannot adequately represent. This recurring theme motivates the uncertainty representations of Section 4 and the integrity-monitoring requirement of Section 4.6.

### 3.4. Automotive Radar: RF Physics and System Implications

Automotive radar operates predominantly in the 76–81 GHz band using FMCW waveforms. Its primary measurements are range and radial velocity, obtained from the beat frequency and Doppler components of the received signal, while azimuth (and, in four-dimensional systems, elevation) is inferred via digital beamforming across physical or MIMO virtual arrays [12,13]. A fundamental waveform design relation is range resolution ΔR ≈ c/(2B), where B is the chirp sweep bandwidth, which ties waveform parameters directly to geometric resolution in tracking and detection. Radial velocity resolution is similarly governed by Δv ≈ λ/(2T), where λ is the RF wavelength and T the coherent processing interval. Angular resolution is constrained by aperture length and the number of independent virtual elements [12,13].

The emergence of 4D imaging radar represents a significant architectural advance: large MIMO arrays with an elevation aperture produce point-cloud-like representations with range, azimuth, elevation, and velocity per detection, enabling object-level 3D scene reconstruction that was previously exclusive to LiDAR [13]. Production-grade examples illustrate the trajectory. Continental’s ARS548 reportedly synthesizes 12 transmit × 16 receive = 192 virtual channels with digital beamforming, achieving roughly 1° azimuth and 2° elevation angular resolution at ranges to about 300 m [55]. The Arbe Phoenix chipset reaches an even larger virtual aperture, targeting sub-1° resolution. On the algorithmic side, RadarOcc [56] demonstrates that 4D radar tensors can serve as first-class inputs for 3D occupancy prediction, motivated by the robustness limitations of camera and LiDAR pipelines in adverse weather conditions. Ghost-target suppression in MIMO configurations exploits the DOD ≠ DOA criterion to identify detections that cannot arise from a single physical scatterer, providing a physics-grounded integrity check [57]. Next-generation waveforms, phase-modulated continuous-wave (PMCW) for interference robustness, OFDM for joint communication and sensing, and OTFS for high-Doppler resilience, position radar as an increasingly capable primary sensing modality rather than a backup [58]. Beyond perception, FMCW automotive radar can serve as an ego-motion sensor under GNSS degradation: multi-level integrated navigation architectures fuse FMCW radar with magnetometer, reduced-inertial sensor systems, and GNSS to maintain bounded position error throughout outages and signal manipulation [59].

### 3.5. GNSS, IMU, and Odometry: Localization Sensors and Fusion Synergy

GNSS receivers measure pseudorange and carrier-phase observables from multiple constellations (GPS, GLONASS, Galileo, and BeiDou). Standard single-frequency positioning achieves 2–5 m accuracy under open sky; Real-Time Kinematic (RTK) and Precise Point Positioning (PPP) achieve centimeter-class accuracy by resolving integer carrier-phase ambiguities, with PPP-RTK/INS tight coupling demonstrating sub-0.2 m horizontal accuracy at >96% availability in urban canyons [15,60,61,62] and complementary perspectives (implementation, review, and survey) [60,61,62,63]. A recent comprehensive PPP review [62] underscores that achievable accuracy is highly sensitive to correction-stream availability and convergence time, which is itself the primary motivation for tight coupling with IMUs and for the integrity-monitoring framework in Section 4.6. The dominant GNSS failure mode in urban environments is multipath and non-line-of-sight (NLOS) reception: signals reflected from building facades traverse longer paths than the direct signal, introducing positive pseudorange biases of meters to tens of meters. These errors are hazardous because they satisfy nominal carrier-to-noise and signal-geometry checks, making them difficult to detect without multi-constellation redundancy or integrity monitoring [15,63]. Receiver Autonomous Integrity Monitoring (RAIM) and Advanced RAIM (ARAIM) provide statistical consistency tests across redundant observations and compute protection levels and worst-case position-error bounds at specified probabilities, which can be directly used by planning algorithms as localization safety margins [15].

**Figure 3:**
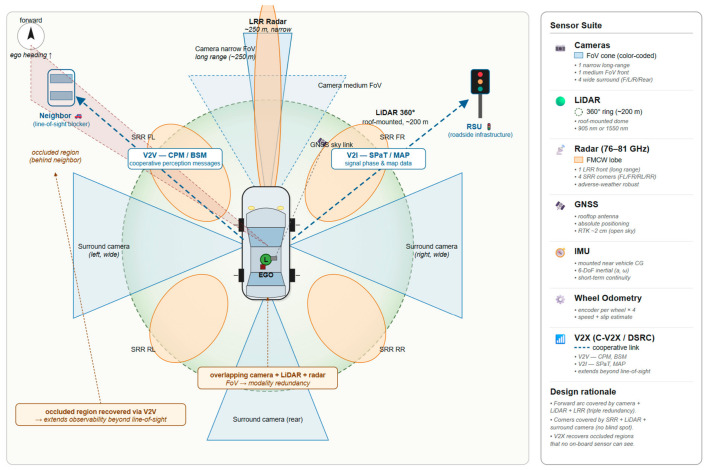
Automotive sensor placement showing forward and surround cameras, roof and bumper LiDAR, front and corner radars, GNSS antenna, IMU, encoders, and V2X links. Field-of-view cones are color-coded by sensing modality as indicated by the in-figure legend (cameras, 360° LiDAR ring, radar lobes), and blue dashed lines denote cooperative V2X links (V2V and V2I) extending observability beyond line-of-sight.

IMUs measure specific force and angular rate at high update rates (100–1000 Hz), enabling pose tracking between GNSS updates and through outages. Pose integration propagates gyroscope and accelerometer biases as quadratically growing position errors and linearly growing velocity errors in the absence of external aiding [14]. Allan variance analysis characterizes bias instability, angle random walk, and rate random walk, determining how long useful dead-reckoning can be maintained. Wheel odometry provides relative displacement increments complementing the IMU at low speeds; its failure modes, wheel slip under braking or on low-friction surfaces, tire-radius changes, and steering-bias errors, produce non-Gaussian, scenario-dependent errors requiring slip detection rather than pure statistical filtering [14,16,34]. The localization robustness principle is that GNSS, IMU, and odometry are complementary in their failure modes: GNSS provides bounded but occasionally biased absolute corrections; IMU provides high-rate increments that grow unboundedly without correction; odometry provides low-latency displacement with scenario-dependent reliability. A well-designed fusion architecture exploits these complementary behaviors while continuously monitoring each modality for integrity violations, ensuring that a biased GNSS fix is not fused as if it were correct [15,34,59,64]. Figure 3 illustrates typical automotive sensor placement and cooperative V2X links; Table 1 summarizes sensor comparison across measurement physics and observability.

## 4. Uncertainty and Error Modeling

Section 3 showed that each sensing modality has a characteristic error structure dictated by physics. This section translates those error structures into formal uncertainty representations. It explains why consistent propagation across the autonomy stack, rather than local confidence scores, is the foundation on which Section 6 (fusion) and Section 10 (planning) must rest. The unifying thread is integrity: sensing, fusion, and decision-making layers share a common obligation to bound the probability of hazardously misleading information at each module boundary.

### 4.1. Error Sources: Random, Systematic, and Environment-Induced

Sensor errors fall into three categories with distinct statistical and system-level signatures. Random errors are zero-mean, high-frequency fluctuations, including thermal noise, photon shot noise, and quantization noise, typically modeled as Gaussian or mildly heavy-tailed and well handled by classical filters. Systematic errors are biases and miscalibrations that persist across measurements: extrinsic calibration drift, tire-radius mismatch, IMU bias, and GNSS multipath. These are hazardous precisely because they are invisible to filters that assume zero-mean residuals, thereby driving the need for explicit bias states and cross-sensor consistency checks. Environment-induced anomalies are event-like, often non-Gaussian error modes triggered by operating-condition transitions: LiDAR backscatter in fog, camera glare, GNSS NLOS in urban canyons, and wheel slip on ice. These cannot be absorbed into Gaussian noise models and instead require event-detection logic, robust estimation (Huber losses, graduated non-convexity), or explicit switching between nominal and degraded measurement models.

### 4.2. Calibration and Synchronization as First-Order Uncertainty Drivers

Extrinsic calibration, the geometric transform between sensor frames, and temporal synchronization, alignment of sensor timestamps to a common clock, determine whether measurements from different modalities can be fused consistently. A 1° rotational calibration error between camera and LiDAR produces a 1.75 m lateral displacement at 100 m range (from simple trigonometry (100 × tan(1°) ≈ 1.75 m), potentially misassociating objects across modalities; a 10 ms timing error at highway speed (30 m/s) produces a 0.3 m longitudinal discrepancy that can break cross-modal association entirely. Calibration and synchronization are, therefore, first-order safety-relevant states rather than one-time engineering steps [59,60]. Online self-calibration via motion-based extrinsic estimation, target-free LiDAR–camera alignment [65,66], and factor-graph-based IMU bias co-estimation [34,67] enables these parameters to be observed as latent states and continuously monitored. Time synchronization across heterogeneous sensors is increasingly governed by IEEE 802.1AS (gPTP) [68], a profile of IEEE 1588 optimized for Time-Sensitive Networking, which achieves sub-microsecond end-to-end synchronization over short network diameters, essential for L3/L4 systems where sensor data must be temporally aligned for BEV projection.

### 4.3. Uncertainty Representations: Four Tiers by Theoretical Guarantee

A sensor-centric survey benefits from a taxonomy of uncertainty representations organized by the strength and nature of their guarantees, since different safety-case claims require different mathematical foundations.

Tier 1: frequentist calibration. Temperature scaling, Platt scaling, and isotonic regression recalibrate classifier outputs so empirical accuracy matches predicted confidence. These methods are computationally cheap and compatible with deployed networks, but they provide no guarantee outside the calibration distribution, a fundamental limitation under SOTIF-triggering conditions.

Tier 2: Bayesian approximation. MC Dropout, deep ensembles, and variational Bayesian neural networks capture epistemic uncertainty by sampling from an implicit posterior over model parameters [18,69,70]. Kendall and Gal formalized the decomposition into aleatoric (irreducible sensor noise, captured via heteroscedastic variance outputs) and epistemic (model ignorance) components, with both forms improving depth regression and segmentation on standard benchmarks [18]. A recent T-ITS review [29] evaluates UQ methods across practicability, robustness, accuracy, scalability, and efficiency, finding that deep ensembles offer the best accuracy–robustness trade-off but at a high computational cost. In contrast, single-pass methods are preferred for real-time deployment.

Tier 3: evidential and belief-function methods. Evidential deep learning [38,71] and predictive prior networks [72] represent class probabilities as a Dirichlet distribution, with the total evidence directly quantifying epistemic uncertainty. Random-Set Neural Networks (RS-NN), published at ICLR 2025 [73], predict belief functions from Dempster–Shafer theory rather than probability vectors, using distributions over the power set of classes; epistemic uncertainty is encoded via the size of the credal set. RS-NN reportedly outperforms both Bayesian methods (LB-BNN, FSVI) and ensemble methods on OOD benchmarks while requiring only a single forward pass, making them an attractive single-pass option for safety-critical deployment, particularly recent driving-specific instantiations such as Durasov et al.’s evidential 3D-detection framework [74], which transfers Tier 3 from classification to the regression heads that the Section 10.2 collision-risk bound directly consumes.

Tier 4: distribution-free conformal prediction. Conformal prediction [20] provides finite-sample coverage guarantees without distributional assumptions by constructing prediction sets calibrated to a user-specified miscoverage level on an exchangeable calibration set. This tier is particularly relevant to ISO/PAS 8800 compliance because it produces statistically verifiable performance envelopes that are robust under mild covariate shift and that map directly to safety-case evidence. Recent conformal extensions for 3D occupancy prediction [75] and uncertainty-aware 3D object detection have begun to bridge the gap between theoretical guarantees and automotive deployment.

As summarized in Table 2, the safety-critical implication of this taxonomy is that no single tier is sufficient for an L4 deployment. A Tier-1-only stack would reproduce the silent-failure cascade of Section 2.2, because temperature scaling cannot detect the OOD object class that initiates such a chain. A Tier-2-only stack improves epistemic awareness but pays a latency cost incompatible with the 100–200 ms control loop (Section 10.4) unless ensembles are isolated on a dedicated compute path. Tier 3 single-pass evidential methods recover the latency budget but require evidence-statistic calibration validated on the deployment ODD. Tier 4 conformal prediction provides the only finite-sample coverage guarantee suitable as ISO/PAS 8800 [23] safety-case evidence; however, its exchangeability assumption fails under temporal drift, motivating adaptive variants [76]. The practical recommendation that runs throughout this survey is, therefore, a layered stack: Tier 2 or Tier 3 within the ODD for aleatoric/epistemic decomposition, Tier 4 wrapping the final perception decision for auditable coverage, and Tier 1 reserved for cheap recalibration of well-understood classifiers.

### 4.4. Classical Probabilistic and Graphical State Estimation

Classical probabilistic state estimation remains indispensable because its uncertainty propagation is explicit and interpretable, and it integrates cleanly with downstream control loops. The Kalman filter [16,33] (KF) and its nonlinear extensions, Extended KF (first-order Taylor linearization), Unscented KF (sigma-point propagation), and particle filters (sample-based non-Gaussian posteriors), provide the workhorse tracking and localization layer in production systems [16,33]. Factor-graph optimization reformulates multi-sensor state estimation as a sparse nonlinear least-squares problem over a probabilistic graphical model, with frameworks such as GTSAM and iSAM2 enabling the consistent fusion of GNSS observables, IMU preintegration factors, wheel odometry, and visual or LiDAR scan-matching constraints [28,34,77]. Factor graphs naturally accommodate asynchronous measurement arrival, variable-lag smoothing, and marginalization of past states, making them the architecture of choice for long-horizon SLAM. Robust loss functions (Huber, Switchable Constraints, Graduated Non-Convexity) are essential to prevent outlier factors from corrupting the optimized trajectory.

### 4.5. Learned Uncertainty: Aleatoric vs. Epistemic

Deep perception networks can produce learned uncertainty estimates directly as part of their output heads. Kendall and Gal [18] established the standard formulation: aleatoric uncertainty is captured by predicting a per-pixel (or per-object) variance alongside each estimate, trained via Gaussian or Laplacian log-likelihood; epistemic uncertainty is captured through ensemble disagreement, MC Dropout, or evidential heads [38]. In the automotive context, aleatoric uncertainty typically reflects irreducible sensing limits, depth-scale ambiguity in cameras, and range-dependent variance in LiDAR. In contrast, epistemic uncertainty captures distribution shift and OOD exposure. Recent work on 3D occupancy prediction demonstrates that lightweight uncertainty heads can be added to existing BEV detectors with modest parameter overhead while producing OOD detection superior to deep ensembles and MC Dropout under camera corruption [75].

Beyond per-object covariance heads, recent work models interaction-aware predictive uncertainty as a first-class uncertainty modality. Liang et al. [78] propose a transformer–transfer-learning trajectory predictor that emits explicit per-mode covariances over neighbor trajectories, capturing multi-modality that homoscedastic Gaussian heads systematically misrepresent. This is the predictive analog of the perception-level aleatoric–epistemic split: uncertainty here arises not from sensing physics but from the latent intent of surrounding agents. Section 10.2 (see Equations (1) and (2)) shows how these per-mode covariances enter the chance-constrained collision-risk bound as the σ_d term, closing the loop from a learned uncertainty representation in Section 4 to a quantitative planning margin in Section 10.

### 4.6. Error Propagation and the Integrity-Monitoring Requirement

As established in Section 2.2, the central thesis of this survey is that autonomous driving safety depends on *propagated* uncertainty rather than on local confidence scores; without consistent propagation across module boundaries, systems accumulate overconfidence, and the planner operates on point estimates that have lost their provenance [16,18].

**Figure 4:**
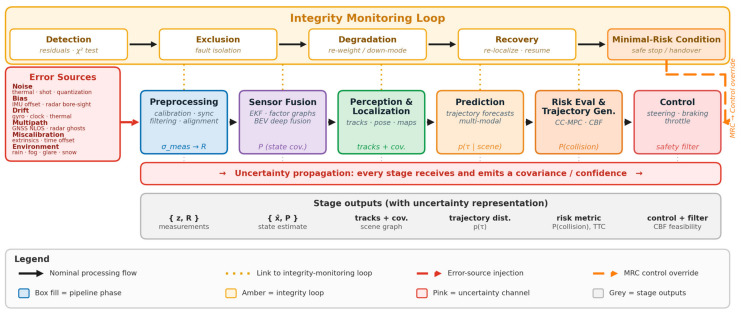
Error propagation pipeline from raw measurement noise to risk-aware control. Each stage (preprocessing, fusion, perception/localization, prediction, risk evaluation, and trajectory generation, control) receives and emits an uncertainty representation, measurement covariance R, state covariance P, track covariances, trajectory distribution p(τ), collision probability, and control-level feasibility. The integrity-monitoring loop above the pipeline performs detection (χ^2^ residual tests), fault isolation, graceful degradation, recovery, and minimal-risk-condition (MRC) activation, keeping downstream safety margins consistent with the calibrated belief about the state. Solid black arrows denote the nominal processing flow; gold dotted connectors link each pipeline stage to the integrity-monitoring loop; the red dashed arrow indicates the injection of the listed error sources into the pipeline; and the orange dashed arrow denotes the minimal-risk-condition (MRC) control-override path.

This propagation requirement motivates integrity monitoring as a first-order system-design concern rather than a post hoc validation step. The GNSS community’s RAIM/ARAIM framework provides the operational template: at each sensor integration step, statistical consistency tests are performed across redundant observations; inconsistencies trigger fault detection and exclusion (FDE); and protection levels, which are bounded worst-case errors at specified probabilities, are computed and propagated to consumers [15,58]. A complementary line of work focuses on correcting (rather than merely detecting) residual pseudorange errors during partial GPS outages in nonlinear, tightly coupled GNSS/INS systems [64], serving as an early sensor-centric demonstration that residual error structure must be modeled rather than assumed away. Generalizing RAIM/ARAIM ideas to multi-modal autonomy requires extending consistency testing to heterogeneous modality pairs (camera vs. LiDAR object lists, GNSS vs. map-matched pose, radar vs. LiDAR tracks), defining multi-sensor protection levels, and integrating OOD detection as the computational mechanism for SOTIF runtime monitoring [7,17,22,62]. In practice, system reliability is governed not by the accuracy of individual sensors but by the consistency of uncertainty modeling across sensing, fusion, and decision-making layers. Figure 4 shows the resulting error-propagation pipeline from sensor physics to risk-aware planning, and Table 3 summarizes sensor uncertainty characteristics and modeling implications for each modality.

## 5. Environmental and Scenario Challenges

The uncertainty representations in Section 4 assume a stable operating envelope; this section shows how real-world environmental conditions stress that envelope and motivate both the fusion architectures of Section 6 and the redundancy strategies of Section 8. Autonomous driving operates within a strictly defined operational design domain (ODD) characterized by weather, illumination, road geometry, traffic norms, and connectivity assumptions. Safety risk increases sharply when the environment departs from the intended functioning envelope, because sensors degrade asymmetrically across modalities and machine-learning models face OOD conditions that invalidate their training priors. This asymmetry, a condition that severely impairs optical sensing, may leave radar unaffected, while GNSS denial in a tunnel has no impact on LiDAR geometry, is the architectural basis for heterogeneous sensor fusion [7,22,69].

### 5.1. Weather, Illumination, and Visibility Degradation

Adverse weather alters sensing not only by reducing signal-to-noise ratios but by fundamentally changing the physical interaction between the environment and sensor hardware. Optical sensing degrades severely under fog, heavy rain, snow, and low-light conditions because image formation depends entirely on scene radiance and contrast. LiDAR experiences severe attenuation, backscatter, and false near-field returns in precipitation and aerosols, with detection probability for distant objects falling below 50% under heavy rain [11,50,51]. Radar is generally the most robust exteroceptive sensor under reduced visibility, but high-intensity rain generates structured clutter at short ranges that demands robust gating and association [69]. Achieving genuine all-weather autonomy requires condition-aware perception pipelines, physical sensor-cleaning systems, and fusion architectures that explicitly recognize partial modality degradation before triggering fallback policies. The fusion system must treat sensor availability and cleaning-system status as latent state variables continuously estimated and propagated into the planner’s risk calculation rather than as binary operational flags.

### 5.2. Urban, Highway, and Rural Operating Contexts

Operational context defines both the sensing challenges and the required planning horizons. Urban environments amplify uncertainty through dense, dynamic occlusions; reflective metallic surfaces that induce RF multipath; and GNSS multipath/NLOS, which can place the vehicle in the wrong lane [15,63]. Highway driving shifts the challenge toward long-range observability and low processing latency: radar’s direct radial-velocity measurement becomes critical for time-to-collision estimation at closing speeds where passive optical depth ambiguity is unacceptable. Rural environments introduce unstructured road boundaries, novel obstacles, and sparse infrastructure, stressing semantic classifiers trained predominantly on urban distributions. These context-specific requirements motivate ODD-aware fusion policies that dynamically adapt sensor weighting and planning conservatism as the ego vehicle transitions between operational contexts. 

### 5.3. GNSS-Denied and Infrastructure-Denied Environments

Tunnels, deep parking structures, and dense urban canyons cause GNSS outages or severe multipath biases, rendering satellite-based absolute positioning unreliable. In these regimes, localization must seamlessly transition from GNSS-primary to IMU/odometry/map-relative fusion, with the architecture actively monitoring integrity to prevent biased absolute updates from corrupting the fused state [15,58,63,64,79]. Adaptive covariance inflation around the GNSS noise model and explicit GNSS outage detection are the primary mitigations; during extended outages, feature-based SLAM relative to HD map landmarks provides a map-anchored alternative that does not depend on satellite observability.

### 5.4. Out-of-Distribution and Edge-Case Scenarios

OOD scenarios arise when operating conditions differ materially from the distribution used to design and train the perception pipeline. OOD events may be environmental (unprecedented lighting, extreme weather), semantic (rare vehicle types, novel animals, erratic pedestrians), or operational (temporary construction patterns, degraded road markings) [17,80]. These situations are particularly hazardous because deep networks may continue to produce high-confidence outputs as model validity declines, thereby creating the silent inference failure mode introduced in Section 3.2. From a SOTIF perspective [22], OOD hazards arise without any component fault: the intended function is computationally insufficient for the encountered scenario. Energy-based OOD detection [19] provides a computationally efficient runtime mechanism: the energy functional of a network’s logit distribution correlates with in-distribution membership, enabling real-time novelty scoring without architectural modification. Conformal prediction [20] provides distribution-free coverage guarantees that remain valid under mild covariate shift, offering a statistically principled mechanism for expanding prediction sets when in-distribution confidence cannot be established. A useful precedent for real-time perception-integrity reasoning over LiDAR point clouds comes from the airborne domain: Massoud and colleagues [81] demonstrate a real-time safe-landing-zone identification pipeline that converts sparse LiDAR returns into a binary safety decision under tight latency constraints. The architectural lesson transfers directly to ground autonomy: a perception module that must signal unsafe/unknown in bounded time is closer in spirit to an integrity monitor than to a conventional detector, and its design must account for compute and memory budgets alongside statistical performance. Table 4 summarizes qualitative sensor reliability across operational scenarios, emphasizing the asymmetric degradation patterns that motivate heterogeneous fusion.

## 6. Multi-Sensor Fusion Architectures

Section 5 showed that no single modality is reliable across the full ODD; this section surveys how heterogeneous measurements are combined into coherent state and scene estimates. A sensor-centric evaluation prioritizes four criteria: uncertainty realism (does the fusion output carry meaningful confidence bounds?), robustness to spatial and temporal misalignment, integrity-monitoring capability, and real-time deployability on automotive embedded hardware [29,82].

### 6.1. Classical and Optimization-Based Fusion

Kalman-family filters remain indispensable for real-time localization and tracking because their explicit covariance propagation produces interpretable confidence estimates that integrate cleanly into risk-aware control loops. The EKF and UKF differ primarily in how they propagate uncertainty through nonlinear motion and measurement models, whereas particle filters represent multi-modal posteriors using weighted samples at a higher computational cost [16,33,67]. Classical filters are robust and well characterized but depend critically on accurate noise modeling and data-association gating: a single miscalibrated noise covariance or missed association can destabilize the filter through innovation buildup. Factor-graph optimization [34] generalizes these ideas to sparse nonlinear least squares over a probabilistic graphical model, enabling the consistent fusion of GNSS observables, IMU preintegration, wheel odometry, visual features, and LiDAR scan matching within a single incremental smoothing pipeline. Its primary challenge is computational cost under dense graph structures; robust loss functions (Huber, Switchable Constraints, Graduated Non-Convexity) are required to prevent outlier factors from corrupting the optimized trajectory.

### 6.2. Learning-Based Multi-Modal Fusion

Bird’s-Eye View (BEV) fusion has emerged as the dominant approach for fusing heterogeneous sensor data because it preserves geometry in a planning-aligned coordinate frame while enabling the combination of camera semantics, LiDAR voxel features, and radar tensors in a single shared grid. BEVFusion [31] addresses the spatial misalignment problem by lifting camera features into 3D via learned depth distributions (Lift-Splat-Shoot [83]) and splatting them into a BEV grid alongside voxelized LiDAR features, using an optimized BEV-pooling kernel that achieves an order-of-magnitude latency reduction in the view-transformation bottleneck. BEVFormer [32] adds temporal self-attention that recurrently fuses historical BEV representations via ego-motion warping, providing implicit temporal alignment without nanosecond-level hardware synchronization. The deeper analytical point is that BEV is not merely a modeling convenience: it reduces the coordinate-reconciliation burden that otherwise migrates into brittle, calibration-dependent point-to-pixel association.

TransFusion [84] develops the same critique from a complementary angle, replacing hard geometric point-to-pixel projection with a soft-association mechanism in which a two-layer transformer decoder generates initial proposals from LiDAR BEV features and then attends from object queries to image features, adaptively focusing on informative image regions while down-weighting corrupted or misaligned ones. The robustness consequence is the critical property for safety-critical fusion: when image quality drops or extrinsic calibration drifts, attention weights migrate toward LiDAR rather than producing spurious cross-modal associations, so the architecture degrades rather than fails under partial sensor compromise.

A second axis of recent progress targets the quadratic sequence complexity of transformer self-attention through state-space models (SSMs). The Mamba family [85] frames sequence modeling as a selective state-space recurrence with linear-time decoding, and driving-relevant variants apply this to motion forecasting and end-to-end planning. Trajectory Mamba [86] replaces self-attention with a selective SSM in a forecasting decoder, reducing parameter count and FLOP cost relative to transformer baselines while remaining competitive on Argoverse 2; Mamba-3 [87] refines the discretization, introduces complex-valued state updates, and adds a multi-input multi-output formulation that improves arithmetic intensity during decoding. The right framing for a sensor-centric survey is directional rather than promotional: SSMs offer a compute-aware path to long-horizon temporal fusion that is attractive precisely because automotive deployment is memory- and latency-bound, but their robustness, calibration, and integrity-monitoring behavior under adverse conditions remain open and must be validated against the same criteria applied to BEV transformers.

### 6.3. Fusion Strategies: Early, Mid, and Late Fusion

Fusion strategies are categorized by where data is combined within the pipeline, each representing a different point on the information-richness-versus-robustness trade-off. Early (data-level) fusion concatenates raw or pre-processed measurements before feature extraction, retaining maximum inter-modal correlations but requiring precise calibration and temporal alignment [82]. Mid-level (feature-level) fusion combines learned features extracted independently from each modality, balancing information richness with computational tractability; BEV architectures operate at this level and represent the current state of the art [31,32]. Late (decision-level) fusion combines independent object hypotheses or tracks produced by per-modality detectors, offering greater robustness to moderate misalignment and graceful degradation, at the cost of discarding synergistic cross-modal correlations. Late fusion remains preferred in safety-critical fallback modes where calibration integrity cannot be guaranteed.

### 6.4. Resilient Fusion and Integrity Monitoring

Resilient fusion architectures explicitly manage sensor failure rather than assuming nominal sensor availability. The Mixture of Multi-Modal Experts (MoMEs) [88] is a representative example whose design principle matters more than any single benchmark number: it deploys three parallel expert decoders, camera-only, LiDAR-only, and fused, and an Adaptive Query Router that evaluates per-query feature quality and routes each object query to the expert most appropriate for the current modality state. Under LiDAR degradation, queries are routed toward the camera expert; under camera dropout, routing shifts to the LiDAR expert; under nominal conditions, the fused expert dominates. Reported evaluations on the nuScenes-R robustness benchmark indicate that this graceful-degradation property produces more stable performance under LiDAR beam reduction, camera drop, and limited field-of-view than monolithic fusion baselines [88]. The lesson for safety-critical deployment is structural rather than numerical: a fusion architecture that decouples modality dependencies and routes based on feature quality can explicitly signal degraded states to the integrity monitor and the planner, rather than silently producing overconfident outputs when one of its inputs has failed.

**Figure 5:**
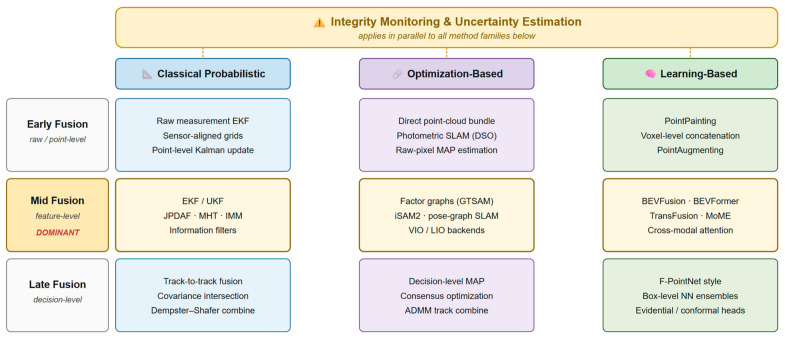
Taxonomy of multi-sensor fusion architectures organized by data-fusion level (early/mid/late) and algorithmic family (classical probabilistic, optimization-based, learning-based). Representative classical methods include EKF/UKF filters, JPDAF/MHT/IMM trackers, factor-graph and iSAM2 SLAM, and track-to-track fusion; representative learning-based methods include PointPainting, BEVFusion, BEVFormer, TransFusion, MoME, and conformal/evidential heads. Integrity monitoring and uncertainty estimation are applied in parallel across all method families, providing the calibrated confidence that each downstream safety argument requires. Column colors denote the algorithmic family (blue: classical probabilistic; purple: optimization-based; green: learning-based); the gold-highlighted row marks the currently dominant mid-level (feature) fusion stage; and gold dotted connectors indicate that integrity monitoring and uncertainty estimation apply in parallel to all families.

Integrity monitoring overlays all fusion families to enforce safety. The RAIM/ARAIM-derived integrity template and its generalization to cross-modal consistency testing and multi-sensor protection levels are developed in Section 4.6 and are not restated here; the point specific to fusion is that every architecture family in this section must expose calibrated residuals to that monitoring layer rather than emit silently overconfident outputs when a constituent modality degrades [7,22,62]. Figure 5 illustrates the fusion architecture taxonomy and representative classical versus learned fusion pipelines; Table 5 provides a comparative summary across fusion level, uncertainty handling, core strengths, and limitations.

## 7. Safety, Reliability, and Standards

The fusion architectures of Section 6 must operate within a safety framework that translates their technical properties into auditable evidence. This section maps sensing and fusion mechanisms onto the principal functional safety, SOTIF, AI safety, safety case, and regulatory standards, emphasizing the sensor-centric interpretation of each: what the standard requires of perception and which architectural mechanism satisfies that requirement. Autonomous driving is a safety-critical cyber–physical system in which sensors are the primary root cause of many high-impact hazards, perception errors, unobserved obstacles, localization divergence, timing violations, and miscalibrations translate directly into unsafe kinematic plans [21,22,25,26].

### 7.1. ISO 26262: Functional Safety and ASIL

ISO 26262 [21] provides the foundational functional safety lifecycle for automotive electrical and electronic systems, decomposing system-level safety goals into hardware and software requirements via Automotive Safety Integrity Level (ASIL) assignments (A–D, with ASIL-D the highest). ASIL assignment is driven by the severity, controllability, and exposure of each identified hazardous event: camera failure during a highway overtaking maneuver may receive ASIL-C or ASIL-D; GNSS outage on a pre-mapped route may receive lower ASIL given map-relative fallback availability. From a sensor-centric standpoint, ISO 26262 mandates systematic analysis of hardware failure modes, sensor dropouts, processor bit flips, timing violations, and requires that safety mechanisms (redundancy, monitoring, fallback) achieve a quantified diagnostic coverage sufficient to reduce residual risk. The safety concept must trace from the vehicle-level hazard through the perception architecture to the individual sensor’s failure mode and the monitoring mechanism that detects it.

### 7.2. ISO 21448 (SOTIF) and Performance Insufficiency

ISO 21448 SOTIF [22] addresses hazards that arise without any component fault, purely because the intended function is insufficient for the encountered scenario. SOTIF is structured around four scenario regions: known safe, known unsafe, unknown safe, and unknown unsafe. The principal challenge for autonomous perception is Region 4, unknown, unsafe scenarios where neither designers nor the testing regime have identified the hazard. OOD detection mechanisms (Section 5.4) serve as the computational mechanism for runtime SOTIF monitoring: detecting that the current scenario lies outside the system’s validated envelope and triggering the appropriate mitigation [7,22]. The SOTIF process mandates iterative scenario analysis, including adverse weather, unusual object classes, edge-case intersection geometry, and complex occlusion, with each identified triggering condition requiring either a design-level mitigation or a validated detection-and-response mechanism. This motivates the ODD-specific reliability analysis of Section 5 and the uncertainty quantification methods of Section 4.3.

### 7.3. ISO/PAS 8800: AI Safety in Road Vehicles

ISO/PAS 8800 (published December 2024) [23] extends ISO 26262 and SOTIF specifically to AI-based systems in road vehicles, providing a consensus specification for managing the non-deterministic risks of machine learning, data bias, distribution shift, and performance degradation in scenarios not represented in training data, building on early treatments of safely using machine learning in automotive systems [89]. The standard defines an AI safety management lifecycle that integrates with the traditional V-model, prescribes methods for generating safety arguments and evidence, and introduces concepts such as the AI safety concept, the AI element out-of-distribution monitor, and AI safety validation. From a sensor-centric perspective, ISO/PAS 8800 requires that uncertainty quantification methods (Section 4.3) be integrated into the AI safety concept as the primary mechanism for demonstrating that a deep perception network’s outputs remain within its validated performance envelope. Conformal prediction [20,75] and evidential deep learning [38] provide the distribution-free and Bayesian uncertainty bounds required to construct statistically verifiable safety arguments. A concrete adoption signal is the August 2025 announcement that SGS-TÜV Saar awarded Geely Auto the world’s first ISO/PAS 8800 process compliance certification [90], indicating that the certification ecosystem for AI safety in road vehicles has begun to shift from specification to audit practice, even if industry-wide maturity remains uneven.

### 7.4. ANSI/UL 4600 and Safety-Case Practice

ANSI/UL 4600 Edition 3 (2023) [24] frames safety evaluation around the construction of a comprehensive evidence-based argument spanning design, validation, and operations. For systems that remove the human driver from the safety loop entirely, UL 4600 requires that the safety case credibly argue [91] that relevant hazards will be mitigated regardless of the specific underlying AI technology, placing the burden of proof on the validity of empirical performance envelopes and uncertainty quantification rather than on algorithm-specific proofs. The Edition 3 update significantly revises the framework for autonomous trucking, addressing extended stopping distances, complex multi-trailer sensor configurations, and refined Safety Performance Indicators, and explicitly adds post-incident behavior requirements (clauses 10.6.6 and 10.6.7 of the standard) that establish how an ADS must record, report, and behave following a safety-relevant event [24]. The architectural implication is direct: SPIs must be measurable at deployment through In-Service Monitoring and Reporting, creating a runtime requirement for uncertainty logging and anomaly detection systems that feed back into the safety case as operational evidence rather than as one-time pre-deployment artifacts.

### 7.5. UNECE Regulatory Framework

The January 2026 UNECE draft Global Technical Regulation (GTR) on Automated Driving Systems [26], adopted by GRVA on 23 January 2026 and pending formal WP.29 adoption later in 2026, represents the first international regulation oriented toward fully unsupervised automated driving. Three provisions are particularly relevant from a sensor-centric perspective. First, a lifecycle Safety Management System (SMS) subject to independent audit governs design, deployment, and post-deployment evolution. Second, mandatory In-Service Monitoring and Reporting (ISMR) requires the operator to surface critical and significant occurrences after deployment, converting fleet experience into evidence that the regulator can act on. Third, a Data Storage System for Automated Driving (DSSAD) mandates timestamped recording of sensing, planning, and control states during automated operation, so that whether the driver or the ADS was in control during a safety event can be reconstructed unambiguously. Crucially, the GTR is outcome-focused: it requires that the ADS perform at least at the level of a competent and careful human driver and be free from unreasonable risk, but it does not prescribe specific sensors or fusion architectures, placing the engineering burden squarely on a credible safety case [91] rather than on a checklist of technologies. UNECE Regulation No. 155 [92], implemented through the ISO/SAE 21434 cybersecurity-engineering process [93], requires a Cybersecurity Management System (CSMS) for type approval, directly affecting V2X trust architectures (Section 5 and Section 9.5). UNECE Regulation No. 156 [94], supported in implementation by ISO 24089:2023 [95] (amended July 2024), enforces a Software Update Management System covering risk management, secure deployment, and configuration tracking, provisions that bear directly on perception stacks where ML models are updated frequently over the air, and where each update is itself a safety-relevant change to the measurement-and-decision pipeline.

### 7.6. Operational Safety Loop: Monitor–Detect–Mitigate–Recover

**Figure 6:**
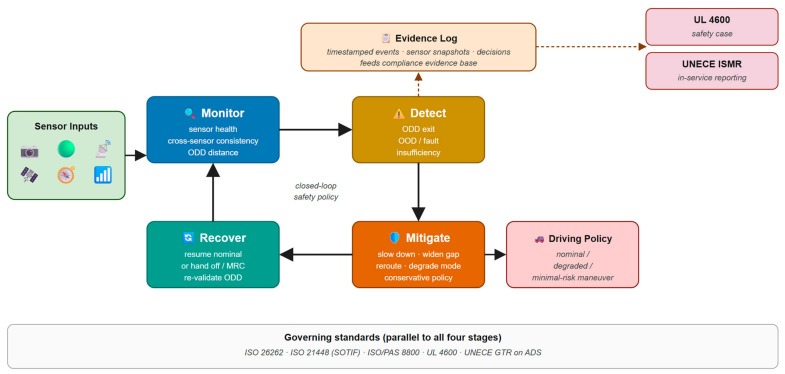
Operational safety loop for runtime hazard handling. The four stages, Monitor (sensor health, cross-sensor consistency, ODD-distance estimation), Detect (ODD exit, OOD exposure, fault, performance insufficiency), Mitigate (slow down, widen gap, reroute, degrade mode, minimal-risk maneuver), and Recover (resume nominal or safe-stop), form a continuous cycle gating the driving policy between nominal, degraded, and minimal-risk regimes. Timestamped events and decisions are written to an evidence log that feeds the UL 4600 safety case and UNECE In-Service Monitoring and Reporting (ISMR) repositories; the entire loop is governed by ISO 26262, ISO 21448, ISO/PAS 8800, UL 4600, and the UNECE GTR on ADSs.Solid black arrows trace the Monitor → Detect → Mitigate → Recover cycle; dashed connectors route timestamped events to the evidence log and onward to the UL 4600 safety-case and UNECE ISMR repositories; box colors distinguish the four loop stages from the sensor inputs (green) and the driving-policy states (pink).

Integrity monitoring signals must be embedded within a closed-loop operational safety policy to function as safety mechanisms rather than passive diagnostics. The four-stage operational safety loop [21,22,23] operates as follows: (1) Monitor sensing health and cross-sensor consistency via signal-level diagnostics, innovation residuals, and ODD boundary distance estimates; (2) Detect ODD exits, performance insufficiencies, sensor faults, and OOD conditions before unsafe outputs propagate to the planner; (3) Mitigate by dynamically modifying the driving policy, reducing speed, increasing following distances, routing away from challenging geometry, or switching to a degraded perception mode; and (4) Recover by re-establishing nominal operation when the anomalous condition resolves, or by executing a minimum-risk stop when recovery is not possible within the ODD. Figure 6 illustrates this closed-loop architecture; Table 6 maps sensor-centric safety requirements to their typical architectural mechanisms across the five standards discussed in this section.

## 8. Redundancy and System Design Trade-Offs

The safety framework of Section 7 ultimately relies on redundancy, diverse, independent measurement and inference paths that break common-cause failure modes. Redundancy in autonomous driving is not a single design decision but a multi-layered resource-allocation problem: additional sensors and compute paths add fault tolerance and diversity but also increase calibration complexity, bandwidth, compute load, thermal constraints, cost, and the probability of common-cause failures due to shared assumptions [21,25]. The optimal strategy depends on the target ASIL level, the ODD breadth, the cost envelope, and the automation level.

### 8.1. Modalities of Redundancy

Sensor redundancy duplicates a single modality, overlapping cameras or dual LiDARs, to tolerate hardware failure and improve spatial coverage, but remains vulnerable to SOTIF-style common-cause insufficiencies if all duplicated sensors share the same observability limits (both cameras blinded by the same glare, both LiDARs attenuated by the same fog). Modal redundancy combines heterogeneous sensing physics, optical camera, RF radar, and laser LiDAR, enabling cross-checks robust to conditions breaking any single modality. Modal redundancy is the primary architectural lever against SOTIF-class environmental hazards because the asymmetric degradation documented in Section 5 ensures that no single environmental condition simultaneously invalidates all sensing paths. Iqbal, Noureldin, and colleagues [96] survey sensor-system schemes for integrated navigation, characterizing their attack surfaces and resilience properties and giving a vocabulary that distinguishes redundancy against random faults from redundancy against systematic insufficiencies. Algorithmic redundancy runs parallel computation paths, a classical EKF tracker alongside a deep BEV network, to reduce common-mode inference risks. Temporal redundancy buffers against transient glitches by integrating multi-frame evidence but requires careful exclusion of stale measurements to prevent outdated data from corrupting the current state.

### 8.2. Fail-Safe vs. Fail-Operational Design

Higher automation levels (SAE Level 4/5) pressure system designs toward fail-operational behavior, maintaining reduced but safe operational capability after a defined fault set, long enough to reach a safer state, rather than fail-safe behavior that triggers an immediate stop. Implementing fail-operational autonomy requires computational isolation between the primary AI pipeline and the safety-monitoring and fallback subsystems. Automotive platforms achieve this through mixed-criticality architectures that employ dedicated lockstep microcontroller safety islands, which execute independent, redundant calculations, ensuring that deterministic fallback logic runs even if high-throughput AI accelerators crash, stall, or violate timing constraints [21,40,97]. The trade-off between fail-safe and fail-operational is ultimately governed by the severity of the fail-safe action in context: an immediate stop may be acceptable on a dedicated highway lane but catastrophic on a dense urban street.

### 8.3. Cost–Performance–Safety Trade-Off Triangle

Every redundancy decision involves a trade-off among three competing objectives: cost (hardware, calibration effort, verification burden), performance (nominal accuracy, latency, throughput), and safety (fault tolerance, OOD handling, regulatory compliance). Consumer ADAS systems prioritize cost, accepting that driver supervision provides the final safety layer. Robotaxi systems prioritize safety, accepting higher unit cost and computing budgets because removing the human driver eliminates the supervision fallback. Highway-only L3 systems occupy a middle ground: they aggressively constrain the ODD to reduce the number of hazardous scenarios that must be handled, allowing a simpler sensor suite than in urban L4. Table 7 summarizes how different deployment targets distribute redundancy across modalities and compute paths.

### 8.4. Real-Time Compute Constraints

Every redundancy and uncertainty-propagation decision is ultimately bounded by the vehicle’s hard real-time budget [98]. Because this constraint and its mixed-criticality implications are analyzed in detail in Section 10.4, we do not duplicate that treatment here and note only that compute cost (latency, throughput, and the verification burden of additional uncertainty-propagation steps) is a first-order term in every redundancy trade-off, not an implementation afterthought. Table 7 summarizes representative redundancy allocations.

## 9. Industry Architectures and Case Studies

The principles of Section 3, Section 4, Section 5, Section 6, Section 7 and Section 8 are most concretely illustrated by how leading deployments have allocated sensors, fusion architectures, and governance mechanisms. Industry deployments demonstrate that there is no universally optimal sensor stack: selection is a function of safety strategy, scalability, cost, and ODD breadth. Examining representative deployments through a sensor-centric lens reveals how physical sensing constraints directly dictate operational postures, redundancy strategies, and regulatory compliance approaches [99,100,101,102].

A note on sources for industry case studies. Because peer-reviewed disclosure of production ADS architectures is sparse, the case-study material in this section necessarily relies on a mixture of company technical communications [99,100,101], regulatory filings and investigation reports [102], industry-standard datasheets [49,51,55], and the limited body of peer-reviewed safety analyses that has emerged from these deployments [91,102,103]. Where peer-reviewed analyses exist, most notably Koopman’s SafeComp/IEEE Spectrum analysis of the Cruise pedestrian-dragging incident [102] and the UL 4600 safety-case literature [91,104], we prefer them. Where no archival source exists, we treat company-reported figures (sensor counts, hardware costs, safety mileage) as the manufacturer’s own claims rather than independently verified facts and so flag them in the text. The architectural lessons we draw from each case study are robust to moderate revisions of the underlying figures, but readers should consult the cited filings and audit reports for the most current verified numbers.

### 9.1. LiDAR-Centric Multi-Modal Autonomy: Waymo

According to publicly available company material [99], Waymo’s sixth-generation driver, introduced for scaled fully autonomous operations beginning February 2026, represents a refined optimization rather than an expansion of the LiDAR-centric philosophy. The reported sensor suite comprises 13 cameras (anchored on a custom 17 MP imager described as “a generation ahead” in dynamic range and low-light sensitivity), 4 LiDARs, 6 imaging radars, and external audio receivers for emergency-vehicle siren detection, with combined coverage extending to roughly 500 m. Notably, the sixth-generation system reduces total sensor count by approximately 42% relative to the fifth generation while reportedly increasing performance, achieved through higher per-sensor capability and in-house rain/snow algorithms in the imaging radars rather than through added redundancy. The system is deployed on the purpose-built Zeekr RT and the Hyundai IONIQ 5 at a per-vehicle hardware cost the company describes as falling below USD 20,000. The architectural lesson is that modal redundancy is being delivered through capability density rather than gross sensor count, with the integrity argument shifting onto per-sensor self-monitoring and the cross-modal consistency layer. These figures are based on publicly available company-reported data and may vary depending on deployment configuration and reporting methodology. Fleet-scale safety data from more than 100 million driverless miles reportedly indicate substantially fewer serious-injury crashes and airbag deployments than the human-comparison baseline; these should be read as company-reported figures rather than independently audited metrics, but they illustrate why the capability-density argument has displaced the brute-redundancy argument in scaled deployments.

### 9.2. Vision-Centric Autonomy: Tesla FSD

Tesla exemplifies an aggressively vision-centric strategy that eliminates rotating LiDAR and conventional automotive radar from the production sensor stack and relies on eight cameras feeding a purpose-built compute platform. As described in publicly available company material [100], Full Self-Driving (FSD) v13 (December 2024) and v14 (October 2025) replaced earlier rule-based control with end-to-end neural networks that combine perception, planning, and control through a temporal-voxel transformer model and a higher-resolution occupancy network. The hardware-generation distinction matters for any safety claim. The earlier HW3 platform offers around 72 TOPS of inference compute and 8 GB of memory; HW4 is widely reported to increase compute substantially (exact specifications not publicly verified), supports 5 MP cameras (versus 1.2 MP on HW3), and reintroduces an optional high-definition forward radar for adverse-weather coverage. FSD v13 and v14 are reported to run only on HW4, leaving the substantial HW3 fleet on an older v12 branch, a fragmentation directly relevant to any fleet-level safety argument. From a sensor-centric perspective, the vision-only architecture shifts the dominant uncertainty from hardware failure modes to epistemic inference uncertainty under OOD conditions. Recent regulatory activity has drawn attention to these concerns. The NHTSA opened Preliminary Evaluation PE25012, as documented in public regulatory filings in October 2025, covering approximately 2.88 million vehicles for FSD-related traffic-safety concerns. A related Engineering Analysis (EA26002) reported that, in multiple crashes, the system “lost track of or never detected a lead vehicle,” including a fatal pedestrian incident [105]. These findings are derived from regulatory reports and should be interpreted within the scope and limitations of ongoing investigations. While not conclusive, they suggest that vision-centric architectures may rely more on inference under challenging conditions, reinforcing the importance of robust integrity-monitoring mechanisms.

### 9.3. Purpose-Built Bidirectional Architecture: Zoox

Zoox’s purpose-built robotaxi adopts a symmetrical bidirectional vehicle architecture with sensor pods at all four corners. The notable sensor-centric feature is the integration of long-wave infrared (LWIR) thermal cameras alongside the conventional camera/LiDAR/radar complement. To the public, Zoox is one of the first commercial AV developers to integrate LWIR in a production sensor suite [101]. LWIR sensors supplied by Teledyne FLIR. LWIR provides pedestrian and animal detection in complete darkness, sun glare, and fog without active illumination, offering an additional modality whose failure modes are largely uncorrelated with those of optical cameras and LiDAR. The company reports a driverless service launch in Las Vegas in September 2025. From an integrity-monitoring perspective, LWIR is interesting precisely because it cross-checks the optical and laser channels in regimes where both can fail simultaneously: night-time pedestrians with low optical contrast, fog where LiDAR experiences backscatter, and sun-glare scenes that saturate visible-light imagers. The architectural lesson generalizes beyond Zoox; additional modalities should be evaluated not by raw performance under nominal conditions but by their *uncorrelated* contribution to the residual risk envelope. 

### 9.4. Hybrid Multi-Modal and Governance: Cruise

Cruise’s deployment trajectory has become a canonical case study for the governance dimension of sensor-centric autonomy. The company operated a heavily sensor-redundant Chevy Bolt AV configuration before its October 2023 pedestrian-contact incident, after which the California DMV suspended its permits, the company halted operations nationwide, and General Motors ultimately wound down the robotaxi program in December 2024. Subsequent regulatory action reportedly included an NHTSA civil penalty of approximately USD 1.5 million and a DOJ deferred prosecution agreement, following the company’s reported admission that it had submitted incomplete information. A safety-engineering analysis of the incident [102] extracts the relevant system-level lessons rather than re-narrating the event: post-collision world-model uncertainty was not handled gracefully; the minimal-risk-condition policy did not adequately account for the possibility of an obscured occupant beneath the vehicle; and the transitions between nominal, degraded, and post-incident modes were not designed as safety-critical operations subject to verification and reporting. For a sensor-centric framework, the Cruise lesson is unambiguous: technical sensing redundancy does not, by itself, guarantee operational safety. Integrity monitoring must be wired into mode transitions; post-incident behavior must be a first-class engineering concern (precisely the territory now addressed by clauses 10.6.6 and 10.6.7 of the UL 4600 Edition 3 standard itself as discussed in Section 7.4); and governance, incident reporting, ODD enforcement, and post-event data preservation are the links between design-time evidence and runtime accountability. The UNECE GTR provisions on SMS, ISMR, and DSSAD discussed in Section 7.5 directly target these failure modes.

### 9.5. Cooperative V2X-Augmented Architectures

V2X-enabled sensing extends the measurement space by importing cooperative object detections, infrastructure sensor data, and beyond-line-of-sight awareness from neighboring vehicles and roadside units. Architectures such as V2X-ViT [41], Where2comm [42], and CoBEVT leverage transformer-based feature aggregation across V2X payloads formatted per SAE J2735 [43] and ETSI CPS [44] standards. Trust assessment is a first-order design requirement: secure message formats (IEEE 1609.2 [106], ETSI TS 103 097 [107]) authenticate transmitter identity but do not guarantee data veracity from faulty or compromised sensors. Subjective logic operators and Bayesian cross-validation against onboard evidence provide a mechanism for dynamically assigning trust scores to incoming cooperative data. The V2X misbehavior-detection problem, identifying “ghost vehicles” broadcasting false collective perception messages, remains an open research challenge, handled in current systems by combining cryptographic authentication with plausibility scoring against onboard perception. Table 8 summarizes the architectural philosophies, sensor suites, robustness levers, risk concentrations, and 2025–2026 deployment status.

## 10. From Perception to Decision-Making

The preceding sections establish that heterogeneous sensors expose the autonomy stack to a structured but irreducible field of uncertainty: photometric noise and depth ambiguity from cameras, weather-induced sparsity and backscatter from LiDAR, multipath and angular ambiguity from radar, and bias-plus-drift coupling in GNSS/IMU/odometry chains. A complete sensor-centric framework must close the loop by demonstrating how those uncertainties propagate through the perception layer, enter the planner’s decision calculus, and ultimately constrain the control commands that act on the physical vehicle. This section formalizes that propagation, articulates the real-time constraints under which it must occur, and shows how OOD detection, integrity monitoring, and risk-aware control translate measurement confidence into safe physical behavior.

### 10.1. The Uncertainty Propagation Chain: From Measurement to Motion

A central insight of the sensor-centric view is that uncertainty cannot be regarded as a property local to any one stage of the pipeline; it is an end-to-end quantity that must remain consistent as it crosses module boundaries. Raw measurement noise is first filtered or learned into a perception belief, object states, occupancy grids, or BEV feature maps, each of which carries an aleatoric component reflecting irreducible sensing limits and an epistemic component reflecting model ignorance [18]. These beliefs are then propagated through tracking, association, and prediction modules, which introduce additional uncertainty due to data-association ambiguity, motion-model mismatch, and intent inference. By the time the planner receives a world model, the original measurement variance has been transformed by a long sequence of nonlinear operators, and the fidelity of the downstream safety argument depends on whether each transformation preserved, inflated, or quietly discarded the underlying uncertainty. Two failure modes dominate this chain. The first is overconfidence collapse, in which a learned perception module emits sharply peaked posteriors that do not reflect epistemic ignorance, causing the planner to accept margins that the sensing layer cannot physically guarantee. The second is uncertainty laundering, in which intermediate modules drop covariance information for computational convenience and re-attach generic noise terms downstream, severing the causal link between physical measurement quality and planner risk. Both pathologies are most dangerous precisely when the system performs well on average benchmarks, because they remain invisible until an OOD or degraded-sensing event reveals that the planner was operating on a fictitious confidence budget.

### 10.2. Risk-Aware Planning Under Uncertainty

Model predictive control (MPC) remains the dominant trajectory-optimization framework in production autonomy because it natively handles vehicle dynamics, actuator limits, and time-varying constraints [108,109]. In its nominal form, however, MPC assumes that obstacle states and free-space boundaries are known with bounded error, an assumption routinely violated under adverse weather, dense urban occlusion, or rare-object encounters. Perception-aware chance-constrained MPC closes this gap by explicitly accounting for the interdependence between sensing quality and control: closer proximity to a target yields higher information gain, which in turn changes whether the chance constraints are satisfied. The Bonzanini–Mesbah–Di Cairano formulation [108] develops this with a multi-stage structure that provides formal guarantees of probabilistic recursive feasibility, illustrating how perception covariance can be folded directly into the optimization rather than treated as an afterthought. Stochastic MPC variants incorporate multi-modal Gaussian-mixture predictions of surrounding-vehicle behavior [110], achieving real-time feasibility at planner-relevant rates. More recent work combines evidential deep learning with distributionally robust optimization so that the controller’s conservativeness is dynamically adjusted by perception confidence [111], a clean operational instantiation of the uncertainty-to-control bridge that this survey advocates.

From perception covariance to planning margins and collision-risk bounds. The transformation that the planner must perform on the perception output is, at its core, a mapping from a state distribution to a deterministic constraint that is safe with high probability. For an obstacle whose lateral position is reported by perception with mean μ_y and variance σ_y^2^ (combining the aleatoric component from sensor noise and the epistemic component from model uncertainty), a chance-constrained planner that requires P(collision) ≤ ε replaces the nominal lateral clearance d_nom by an inflated margin

**Figure 7:**
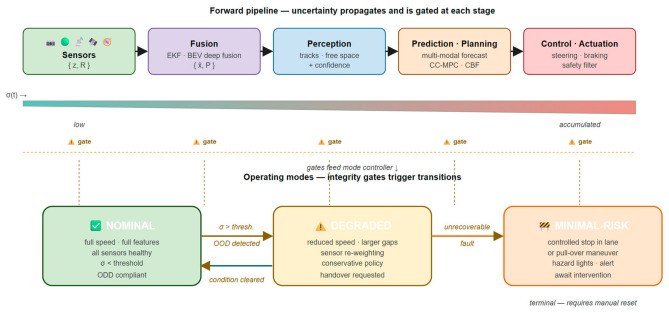
End-to-end uncertainty propagation and mode-switching logic. The forward pipeline (sensors → fusion → perception → prediction/planning → control/actuation) passes a growing uncertainty budget σ(t) across each module boundary; integrity gates at every interface can trigger transitions between nominal, degraded (reduced speed, sensor re-weighting, conservative policy, handover request), and minimal-risk (controlled stop or pull-over) operating modes. Transitions between modes are governed by hysteresis thresholds on OOD scores, innovation residuals, and cross-sensor consistency checks, with the minimal-risk state as the terminal state until manual reset. Box colors encode the operating modes (green: nominal; yellow: degraded; orange: minimal-risk); the horizontal gradient bar depicts the uncertainty budget σ(t) growing from low (teal) to accumulated (red); solid gold and orange arrows denote escalating mode transitions, the teal arrow denotes recovery to nominal, and gold dashed connectors link the per-stage integrity gates to the mode controller.

*d_safe* = *d_nom* + *k*(*ε*) · *σ_y*, (1)

where k(ε) is a quantile factor (e.g., k = 1.96 for ε = 0.025 under a Gaussian assumption, or a Chebyshev/Cantelli bound k = √((1 − ε)/ε) when the distribution is unknown) and σ_y aggregates the per-stage uncertainties propagated from raw measurements through tracking and prediction. Equation (1) is what makes the perception covariance physically actionable: a doubling of σ_y at the perception output causes the planner’s lateral clearance to widen by k(ε) · σ_y meters, directly shrinking the feasible action set. For a longitudinal collision-probability bound under heteroscedastic noise, a useful upper bound is the Cantelli inequality applied to the gap-error random variable Δd = d_actual − d_min:

*P*(*collision*) = *P*(*Δd ≤ 0*) ≤ *σ_d*^2^/(*σ_d*^2^ + *μ_d*^2^), (2)

where μ_d and σ_d are the mean and standard deviation of the gap error under the current sensing regime. Two operational consequences follow. First, sensor degradation (rain, occlusion, calibration drift) inflates σ_d, which directly raises the upper bound on collision probability and forces the planner either to widen d_nom or to lower speed so that the time-to-collision distribution remains within the ε-budget. Second, the same equation gives the ADS designer a quantitative criterion for when to leave nominal mode: if the bound in Equation (2) exceeds the policy’s safety budget, a degraded mode (lower speed, wider gaps) or a minimal-risk maneuver must be triggered, exactly the mode-switching logic of Figure 7. Predictive uncertainty from interaction-aware trajectory forecasters provides the analogous mechanism for surrounding agents: recent transformer–transfer-learning prediction frameworks [78] estimate explicit per-mode covariances over neighbor trajectories, which the planner can ingest as additional terms in σ_d under multi-agent interaction, enabling collision-risk bounds that respect the multi-modal nature of human driving. Reinforcement-learning-based highway and lane-change controllers, reviewed comprehensively in [112,113], offer a complementary policy-level path that, when wrapped in a CBF or chance-constrained safety filter [114], can consume the same uncertainty signals while preserving formal safety guarantees; recent end-to-end reinforcement-learning decision-making on roads with consecutive sharp turns [115] is a concrete instance of this direction in an extreme-geometry regime where perception degradation and vehicle dynamics interact most strongly. In all these cases, the underlying message is identical: perception uncertainty does not merely flag risk; it sizes the safety margins that the controller must enforce.

Formal safety filters provide a complementary deterministic layer atop probabilistic planning. Responsibility-Sensitive Safety (RSS), formalized by Shalev-Shwartz, Shammah, and Shashua [116], encodes a set of human-interpretable longitudinal safety constraints in closed form. The minimum safe longitudinal distance is given by

*d_min* = *v_r ρ* + (1/2) *a_max,accel ρ*^2^ + (*v_r + ρ a_max,accel*)2/(2 *a_max,brake*) − *v_f*^2^/(2 *a_max,brake*) (3)

where v_r and v_f denote the rear (ego) and front vehicle velocities, respectively, ρ is the reaction time, and a_max, accel, and a_max, brake represent the maximum acceleration and braking capabilities. IEEE 2846-2022 [117] generalizes these assumptions by standardizing minimum reasonable safety parameters for automated driving system (ADS) models. Critically, Koopman [39] identifies two key limitations that must be considered in a sensor-centric framework: (i) worst-case parameter assumptions can render RSS overly conservative under nominal traffic conditions, and (ii) mid-maneuver dynamic changes (e.g., sudden transitions from dry to icy road surfaces) are not captured by the closed-form model and must instead be inferred from perception and state estimation.

Control barrier functions (CBFs) provide a control-theoretic alternative for enforcing safety constraints. The canonical CBF-based quadratic program (CBF-QP) computes the control input u by solving

u* = *argmin_u* ||*u* − *u_ref*||^2^ (4)

subject to

*L_f h*(*x*) + *L_g h*(*x*) *u* + *α*(*h*(*x*)) ≥ 0, (5)

which guarantees forward invariance of the safe set

*C* = {*x* ∈ *ℝ^n^*: *h*(*x*) ≥ 0} (6)

Here, h(x) defines the safety function, L_f h(x) and L_g h(x) denote Lie derivatives along system dynamics, and α(·) is an extended class-K function ensuring constraint satisfaction. A recent Annual Reviews in Control tutorial [118] surveys advances in CBF design, including high-order formulations, robustness to disturbances and input constraints, and adaptive parameter tuning.

### 10.3. Out-of-Distribution Detection and System Adaptation

OOD detection is the operational mechanism by which the autonomy stack recognizes that the implicit assumptions of its perception and prediction models no longer hold. Modern approaches range from feature-space distance methods such as the Mahalanobis-distance score [119], which fits class-conditional Gaussians in the penultimate-layer feature space, to energy-based scoring [19], which uses the free-energy functional E(x) = −log Σ_c exp(f_c(x)) of a network’s logits as a theoretically motivated alternative to softmax confidence. Higher-capacity formulations include ensemble disagreement, MC Dropout, and the Random-Set Neural Networks discussed in Section 4.3 [73]. From a sensor-centric standpoint, the value of these methods is not the raw novelty score itself but the mapping from that score to a concrete behavioral adaptation: speed reduction, increased following distance, suppression of high-risk maneuvers, escalation to a degraded operating mode, or initiation of a minimal-risk maneuver. A representative architecture in this direction is AESOP [120], which couples foundation-model-based anomaly detection to a tree of recovery trajectories, so that detection of an unknown/unsafe condition triggers a pre-validated fallback rather than a generic warning. This pattern operationalizes ISO 21448’s “unknown unsafe” region directly: the OOD detector becomes the SOTIF runtime monitor, and the recovery tree provides the verifiable mitigation evidence required by the safety case. This mapping must be designed jointly with the integrity-monitoring loop in Section 7. When a cross-sensor residual check, an innovation gate, or an OOD score crosses its threshold, the planner does not merely receive a warning; it transitions to a different cost function, constraint set, or policy. The transition itself must be verified to be safe under the conditions that triggered it; a planner switching into an aggressive evasive maneuver on degraded perception may be more dangerous than one that decelerates.

### 10.4. Real-Time Constraints and Computational Co-Design

Decision-making under uncertainty is bounded not only by statistical correctness but by the hard real-time budget of the vehicle. A typical perception-to-control loop operates at 10–20 Hz for planning and 50–100 Hz for low-level control, with end-to-end latency budgets of 100–200 ms from photon arrival to actuator command. Within this envelope, every additional uncertainty-propagation step competes for compute against the underlying perception and prediction networks. Co-design across the stack is, therefore, essential: lightweight uncertainty heads on BEV detectors, analytic covariance propagation through tracking, and quadratic-program formulations of safety filters that admit warm-starting all serve to keep the uncertainty-aware loop within the real-time envelope. Hardware safety islands and mixed-criticality architectures provide the deterministic substrate on which this co-design becomes tractable: by isolating the integrity monitor, safety filter, and MRC controller on a lockstep microcontroller separate from the high-throughput AI accelerator, the system guarantees fallback execution even if the primary perception network stalls [40]. Figure 7 makes explicit the central thesis of this section: every arrow in the autonomy pipeline carries not only a state estimate but a calibrated belief about that estimate, and every module is responsible for transforming that belief faithfully. Table 9 operationalizes the figure by mapping each perception/fusion product to its uncertainty representation, planning/control use, and dominant failure mode with corresponding safety action, making clear that no single mitigation strategy suffices (covariance inflation for tracked-object overconfidence, cross-sensor validation for occupancy hallucination, mode-preservation for prediction collapse, hard mode-switching with hysteresis for OOD detection).

## 11. Future Directions and Research Gaps

The sensor-centric synthesis developed across Section 3, Section 4, Section 5, Section 6, Section 7, Section 8, Section 9 and Section 10 surfaces a coherent set of research gaps that are most productively viewed as a single interconnected problem: how to make uncertainty calibrated, observable, and actionable along the entire path from photon to actuator. This section organizes the most consequential gaps under five themes: datasets and benchmarks, cooperative perception and V2X, AI safety and verification, advanced sensing paradigms, and continuous calibration with sensor health monitoring, and it identifies the deployment-relevant questions that remain open for each.

### 11.1. Safety-Critical Datasets and Scenario-Indexed Benchmarks

Public datasets such as nuScenes [121], the Waymo Open Dataset [122], and Argoverse 2 have driven enormous progress in multi-modal perception, but they remain dominated by nominal urban conditions and underrepresent the long-tail events most relevant to safety-critical deployment. The gap is not merely one of volume; it is one of organization. Existing benchmarks measure mean average precision and tracking accuracy on shuffled frames, providing little insight into how a system behaves at the boundary of its ODD. The community needs benchmarks indexed by environmental factors, precipitation intensity, illumination class, GNSS quality, and occlusion density, so that performance can be reported as a function of the operating envelope rather than as a single aggregate number. Two recent developments illustrate the trajectory. The DrivingGen benchmark [123] is an early attempt to evaluate generative driving world models across distribution, perceptual quality, temporal consistency, and trajectory controllability dimensions; its central finding, that no current model dominates all four axes, clarifies what a scenario-indexed benchmark must measure. Separately, Genie 3 [124] is a general-purpose interactive world model that the Waymo team has publicly described as the basis for the “Waymo World Model,” which is used to generate rare edge-case driving environments for training and validation. These should be read as company-described capabilities rather than independently audited benchmarks, but they signal that generative simulation is being pulled into the safety-case toolchain. Calibrated-uncertainty benchmarks remain similarly underdeveloped: no widely adopted protocol exists to measure whether an OOD score or covariance estimate is reliability-calibrated with respect to downstream collision risk.

### 11.2. Cooperative Perception and V2X: Latency, Trust, and Security

V2X communication promises to extend onboard observability beyond line-of-sight, mitigating occlusion-driven failure modes that dominate urban autonomy. The technical foundations, SAE J2735, 3GPP NR-V2X, ETSI TS 103 097, and IEEE 1609.2, are mature [41,42,43,44,79,106,107]. Beyond message exchange, V2X-assisted distributed computing and control frameworks coordinate cooperative maneuvers such as ramp merging by jointly offloading computation and sharing control decisions across connected and automated vehicles and infrastructure [125]. The unsolved problems lie one layer up. Latency variability across the wireless channel creates a moving target for time alignment that deterministic timing protocols cannot absorb; trust in the content of received messages cannot be reduced to authentication of their source, because a cryptographically valid sender may be operating with degraded sensors or compromised software; and the cooperative perception layer must implement plausibility scoring that cross-validates V2X claims against onboard evidence in a way that degrades gracefully when the two disagree. A particularly underdeveloped area is the joint cyber–physical integrity model. Today’s V2X security stack treats authentication, replay protection, and certificate revocation as distinct from the perception integrity monitor of Section 7, even though the failure modes they address are often coupled. Ghost-vehicle attacks, fabricated detections injected into collective perception messages, were systematically studied on the OPV2V cooperative-perception testbed [126], and recent reputation-plus-majority detection schemes, such as CATS [127], have been reported to scale to city-size simulations. The VeReMi dataset [128] remains the standard benchmark for misbehavior detection and provides a community reference point for trust assessment. Unifying these mechanisms with the perception integrity layer into a single trust-and-integrity framework, with shared thresholds and shared mitigation policies, is an open research direction with direct implications for the UNECE R155 CSMS and the 2026 UN GTR on automated driving.

### 11.3. AI Safety: Explainability, Verification, and Robustness

The publication of ISO/PAS 8800 formalized the expectation that AI components in road vehicles will be developed under a lifecycle process analogous to the ISO 26262 V-model, with explicit requirements for data management, training-procedure assurance, and runtime monitoring [8,23,89,110]. Translating that expectation into engineering practice exposes three persistent gaps. First, formal verification of deep neural networks remains computationally intractable at the scale of modern BEV transformers and SSM sequence models, thereby necessitating reliance on statistical testing and runtime monitors, whose coverage arguments are difficult to make rigorous. Second, explainability methods that satisfy regulatory expectations often produce post hoc rationalizations that do not faithfully reflect the network’s internal decision-making process. Third, adversarial and distributional robustness remain active failure surfaces. The most promising research directions change what is being verified rather than how: compositional safety arguments in which a learned perception component is wrapped by a verified runtime monitor with provable false-negative bounds allow formal effort to focus on the monitor rather than the network; conformal-prediction-based uncertainty wrappers [20,75] provide finite-sample coverage guarantees in the presence of distribution shift; and modular safety cases that decompose the autonomy claim into perception, prediction, planning, and monitoring sub-claims align naturally with UL 4600 and ISO/PAS 8800.

A parallel and partly orthogonal direction is the rise of vision–language–action (VLA) foundation models for driving. Recent examples include DrivingGPT, which unifies world modeling and planning under a multi-modal autoregressive transformer; OmniDrive, which packages 3D perception, reasoning, and planning into a single LLM-agent framework; and SafeAuto, which targets safe driving behavior through knowledge-enriched multi-modal foundation models [129]. The sensor-centric question these models raise is unresolved: do they represent and propagate measurement uncertainty in a form downstream safety arguments can use, or do they obscure it behind a single learned policy? Based on current evidence, the answer is the latter, which is why this survey treats VLA models as a promising but not yet safety-mature direction, with integration with integrity monitoring an open problem.

### 11.4. Advanced Sensing Paradigms: RIS, Imaging Radar, and Infrastructure-Assisted Sensing

The next generation of sensing technologies offers the possibility of breaking through current modality-specific limitations. Reconfigurable Intelligent Surfaces (RIS) [130], originally developed for wireless communication, can be repurposed to shape the radar propagation environment and provide controllable non-line-of-sight illumination of occluded regions. Four-dimensional imaging radar, already discussed in Section 3.4, narrows the angular-resolution gap with LiDAR while retaining robustness in adverse weather. Event-based cameras [131], with microsecond-latency asynchronous pixel updates and extreme dynamic range, address the rolling-shutter and HDR limitations of conventional sensors in scenarios with rapid illumination changes. Each of these technologies is mature enough for laboratory deployment but immature with respect to the calibration, time synchronization, and uncertainty modeling tooling that production fusion stacks require. Infrastructure-assisted sensing is the architectural complement: roadside LiDAR and camera installations at intersections, supplemented by edge computing, can provide a stable external reference that is unaffected by vehicle motion or onboard sensor contamination. The research challenge is not the sensing itself but the *federation*, how to fuse vehicle-resident and infrastructure-resident observations under heterogeneous timing, trust, and ownership models without introducing new single points of failure. 

### 11.5. Continuous Self-Calibration and Sensor Health Monitoring

**Figure 8:**
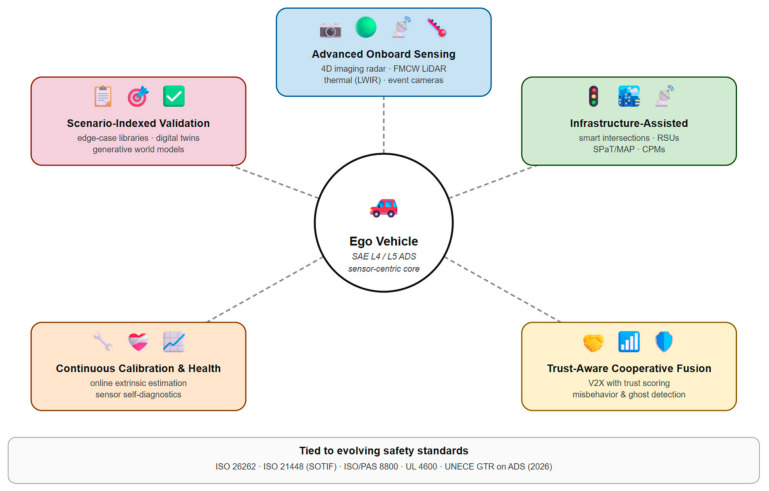
Future sensor-centric autonomy ecosystem around the ego vehicle. Five interconnected directions support the same measurement-to-assurance goal: advanced onboard sensing (4D imaging radar, FMCW LiDAR, long-wave infrared thermal cameras, event cameras); infrastructure-assisted sensing (smart intersections, roadside units, SPaT/MAP and collective perception messages); trust-aware cooperative fusion with V2X misbehavior and ghost-vehicle detection; continuous calibration and sensor-health monitoring (online extrinsic estimation, self-diagnostics); and scenario-indexed validation (edge-case libraries, digital twins, generative world models). All five are tied to the evolving landscape of safety standards, including ISO 26262, ISO 21448, ISO/PAS 8800, UL 4600, and the January 2026 UNECE GTR on ADS. Each peripheral box is color-coded to one of the five research themes, and gray dashed connectors indicate their conceptual linkage to the sensor-centric ego-vehicle core.

Section 4 identified calibration and synchronization as first-order uncertainty drivers, and Section 9 documented the substantial engineering investment production programs make to maintain them. What remains an open research problem is the transition from periodic offline calibration to continuous, observable self-calibration that runs as a first-class component of the autonomy stack. The technical building blocks exist, motion-based extrinsic estimation, target-free LiDAR–camera alignment [65], and online IMU bias estimation through factor-graph smoothing [34], but they have not yet been unified into a calibration health service that provides the planner with a real-time, calibrated estimate of how much it can trust each sensor’s geometry. The same observation applies to physical sensor health: window contamination, fascia damage, internal thermal drift, and partial element failures in MIMO radar arrays all degrade sensing in ways that are, in principle, observable from cross-sensor residuals and reflectivity statistics, but are rarely surfaced as named state variables that the planner can act on. Treating sensor availability and calibration quality as latent states in the fusion graph, with explicit posterior distributions and explicit mitigation policies, is the natural completion of the sensor-centric framework developed in this survey. Figure 8 summarizes the anticipated sensor-centric autonomy ecosystem and emphasizes that the five research themes of this section are not independent agendas but interconnected contributions to a single measurement-to-assurance goal.

### 11.6. Limitations and Scope of This Survey

For credibility as a survey, several limitations of the present treatment should be made explicit. First, several emerging technologies discussed in Section 7.3 and Section 11.3, in particular, vision–language–action (VLA) foundation models such as DrivingGPT, OmniDrive, and SafeAuto, remain too immature for safety-critical deployment to support firm conclusions. Where these models are mentioned, they should be read as plausible research directions rather than as recommended components of an L4 stack; whether they can represent and propagate calibrated uncertainty in a manner usable by the integrity-monitoring layer is at present an open question, and our coverage of them is correspondingly cautious. Second, the regulatory landscape and especially the UNECE Global Technical Regulation on ADSs adopted by GRVA in January 2026 is still in flux at the time of writing; we report the current draft state and expected June 2026 WP.29 adoption, but final provisions may differ from the draft we cite. Third, the industry case studies in Section 9 rely on company technical communications and regulatory filings rather than peer-reviewed disclosures; this limitation is intrinsic to the domain (production architectures are largely proprietary), but it bounds the strength of architectural conclusions that can be drawn from any single deployment. Fourth, the survey is sensor-centric by design and does not provide deep coverage of adjacent areas, driver–vehicle interaction, V2X spectrum policy, energy-efficient eco-driving [132], or generic reinforcement-learning policy synthesis. Where these areas intersect with sensing and uncertainty, we cite representative work, for example, multi-objective deep-reinforcement-learning eco-driving that jointly optimizes driving safety and energy use [132], but we do not attempt comprehensive coverage. Fifth, several quantitative claims about commercial systems (sensor counts, hardware costs, fleet-scale safety statistics) are reported as company-stated values; readers should treat them as the manufacturer’s own claims rather than independently audited measurements. Acknowledging these limitations does not diminish the survey’s framework but clarifies the regime within which its claims are credible.

## 12. Conclusions

This survey has argued that the safety and reliability of autonomous driving systems are bounded, in the final analysis, by what their sensors physically measure, how faithfully the resulting uncertainty is represented and propagated, how robustly heterogeneous measurements are fused into a coherent world model, and how rigorously the residual uncertainty is translated into conservative, verifiable behavior. The five pillars of the framework, sensing physics, uncertainty modeling, fusion integrity, safety assurance, and risk-aware planning, are not independent layers to be optimized in isolation. They form a single causal chain in which a weakness at any point silently undermines every downstream guarantee.

The technical synthesis can be stated compactly. Cameras deliver dense semantics but infer geometry, making them powerful under nominal conditions and silently fragile under OOD exposure. LiDAR anchors metric structure but degrades asymmetrically under precipitation, backscatter, and contamination, with wavelength selection (predominantly 905 nm) trading detector cost against eye-safety budget and weather propagation. Radar provides direct range and Doppler velocity with all-weather robustness, and 4D imaging radar is rapidly closing the angular-resolution gap with LiDAR. GNSS, IMU, and odometry combine to deliver continuous global pose, but only when fused under integrity monitors that prevent biased updates from corrupting the state estimate. Classical Bayesian filters, factor-graph smoothers [34,77,133], BEV and transformer-based learned fusion, resilient expert-routing architectures such as MoME, and emerging state-space architectures such as Mamba-3 each contribute distinct strengths, and each must be wrapped in explicit uncertainty handling and integrity monitoring to be deployable in safety-critical contexts. The standards landscape, ISO 26262, ISO 21448, ISO/PAS 8800, ANSI/UL 4600, the UNECE R155/R156/R157 family, and the January 2026 UN GTR on automated driving, has converged on the same conclusion from the regulatory side: AI-enabled autonomy must be engineered as a measurement-and-assurance system, with traceable evidence linking sensing performance to operational behavior.

The practical implications for system designers are concrete. Uncertainty must be a first-class output of every perception module, calibrated against downstream risk rather than against intermediate accuracy metrics. Calibration and synchronization must be treated as continuously observable states, not as one-time engineering artifacts. Integrity monitoring must be wired directly into planning and control through explicit mode-switching logic with verified hysteresis, not bolted on as a passive diagnostic. Redundancy must be designed to break common-cause failure modes, which means modal and algorithmic diversity rather than mere duplication. Risk-aware planning, through perception-aware chance-constrained MPC, RSS envelopes, and CBF safety filters, must receive uncertainty-inflated margins rather than point estimates from the perception layer. And the entire stack must be co-designed with the real-time and mixed-criticality constraints of the underlying compute platform, so that fail-operational behavior remains deterministic even when the primary AI accelerator is degraded.

The distinctive contribution of this survey is the explicit linkage among these five pillars under a single sensor-centric throughline. Where prior work has tended to treat perception, fusion, uncertainty, safety, and planning as separable research communities, the thesis here is that safety emerges from their coherence, not from their individual accuracy. The remaining gaps, scenario-indexed benchmarks, joint cyber–physical integrity, formally verified runtime monitors, RIS- and infrastructure-assisted sensing, continuous self-calibration with explicit health states, and the integration of VLA foundation models with integrity monitoring, are well defined and tractable, even if individually difficult. Closing them will not by itself produce ubiquitous Level 5 autonomy, but it will produce something arguably more valuable: deployable Level 4 systems whose safety arguments are credible, whose failure modes are observable, and whose behavior under stress is predictable. That, rather than any single algorithmic breakthrough, is the path along which sensor-centric autonomous driving will scale from constrained pilot deployments to broad real-world use.

## Figures and Tables

**Table 1:** Sensor comparison: measurement physics, direct versus inferred quantities, key strengths, and dominant limitations for camera, LiDAR, radar, GNSS, IMU, and odometry.

Sensor	Active/Passive	Physical Measurement	Direct vs. Inferred	Key Strengths	Dominant Limitations
Camera	Passive	Pixel irradiance/colour	Direct: 2D image Inferred: depth, scale, velocity	Dense semantics; high angular resolution; low cost	No metric depth; illumination/weather sensitivity; compute-intensive inference
LiDAR	Active	ToF/FMCW range returns	Direct: sparse 3D depth Inferred: object class, velocity (ToF)	Precise metric geometry; map-relative localization; FMCW gives per-point velocity	Weather attenuation and backscatter; sparse at range; contamination; FMCW manufacturing complexity
Radar	Active	76–81 GHz FMCW beat freq. + Doppler	Direct: range and radial velocity Inferred: azimuth angle, object class	All-weather robustness; direct velocity; low cost; emerging 4D imaging	Coarse angular resolution; multipath/ghost targets; clutter; limited semantics
GNSS	Passive	Pseudorange and carrier phase	Direct: observables Inferred: global pose and time	Global reference frame; map anchoring; PPP-RTK sub-0.2 m accuracy	Multipath/NLOS; outages in urban canyons/tunnels; correction-service dependence; integrity monitoring essential
IMU	Active	Specific force and angular rate	Direct: inertial increments Inferred: pose via integration	High-rate; self-contained; bridges GNSS outages; orientation continuity	Bias drift; scale-factor error; vibration sensitivity; unbounded position error without aiding
Odometry	Active	Wheel rotation increments	Direct: incremental displacement Inferred: planar trajectory	Low cost; low latency; complements IMU at low speed	Slip/ice failures; tire-radius and steering-bias errors; unbounded drift without correction

**Table 2:** Assumptions, limitations, and safety-critical suitability of the four uncertainty tiers for autonomous-driving perception. Reference [74] in row Tier 3 denotes Durasov et al.’s evidential 3D-detection framework; reference [76] in row Tier 4 denotes adaptive conformal prediction under distribution drift.

Tier	Method Examples	Assumption and Dominant Failure mode	Safety-Critical Suitability
Tier 1, frequentist calibration	Temperature, Platt, isotonic scaling	Test ≡ calibration distribution; silent miscalibration under covariate shift (SOTIF)	In-ODD baseline only; not trustworthy at ODD boundaries
Tier 2, Bayesian approximation	MC Dropout; deep ensembles; VBNNs [18,69,70]	Variational family/ensemble ≈ true posterior; underestimates epistemic UQ on novel inputs; high latency cost	Strong in-ODD with mixed-criticality compute (Section 8)
Tier 3, evidential/belief-function	EDL [38,71]; prior networks [72]; RS-NN [73]; 3D-detection EDL [74]	OOD leaves a recognizable evidence/credal signature; regularizer-sensitive; empirical (not finite-sample) coverage	Single-pass; evidence statistic must be ODD-calibrated
Tier 4, conformal prediction	Split/adaptive conformal [20]; conformal occupancy [75]	Calibration and test data exchangeable; fails under temporal drift; sets inflate under heavy shift	Best fit for ISO/PAS 8800 [23] safety case; adaptive variants [76] under drift

**Table 3:** Sensor uncertainty characteristics and implications for uncertainty modeling for each sensing modality.

Sensor	Typical Random Error Distribution	Systematic Error Mode	Environment-Driven Anomalies	Uncertainty Modeling Implication
Camera	Heteroscedastic photometric noise; blur variance	Intrinsic drift; rolling shutter	Glare; fog; rain; lens contamination	Depth and velocity inferred, aleatoric uncertainty is depth-scale ambiguity; epistemic uncertainty is critical for OOD illumination/weather
LiDAR	Range variance increasing with distance; sparsity effects	Extrinsic/scan-timing bias; window drift	Fog/rain attenuation; backscatter; contamination	Mixture/heavy-tail noise models preferred; robust estimation (Huber, GNC) valuable; FMCW enables richer velocity uncertainty
Radar	Range/velocity variance; angular noise	Array miscalibration; interference bias	Multipath ghost targets; rain clutter	Structured clutter models required; gating and robust association; ghost-target probability should be propagated
GNSS	Processed noise; DOP-dependent	Multipath/NLOS bias	Urban canyon blockage; jamming	Outlier-prone, integrity monitoring (RAIM/ARAIM) is the primary defense; protection levels define planning safety margins
IMU	High-rate white noise	Bias drift; scale-factor; temperature	Vibration; shock	Integration causes rapid error growth, bias uncertainty must be co-estimated; Allan variance characterization essential for filter tuning
Odometry	Quantization; small random slip	Tire-radius/steering bias	Slip on ice/gravel	Scenario-dependent confidence required; slip detection as a discrete fault event rather than a continuous Gaussian uncertainty

**Table 4:** Scenario-driven sensor reliability (High/Medium/Low) and dominant safety risk drivers for nominal, night, rain, fog, snow, urban-canyon, highway, and tunnel contexts.

Scenario	Camera	LiDAR	Radar	GNSS	IMU	Odometry	Dominant Risk Drivers
Clear day/open sky	High	High	High	High	High	High	Calibration drift; rare OOD objects; compute limits
Night/heavy glare	Low–Med	High	High	High	High	High	Camera contrast loss; optical glare; LED flicker
Heavy rain	Med–Low	Med–Low	Med–High	Medium	High	Medium	Optical attenuation; LiDAR backscatter; radar clutter; traction slip
Dense fog/haze	Low	Med–Low	High	Medium	High	High	Optical absorption; scatter; severe visibility loss
Snow/slush	Med–Low	Low	Med–High	Medium	High	Low–Med	LiDAR false returns; severe odometry slip; object classification
Dense urban canyon	Medium	High	High	Low	High	High	GNSS multipath/NLOS; dynamic occlusion; RF multipath
Highway (high speed)	High	High	High	High	High	High	Latency constraints, long-range TTC accuracy, and closing speed
Tunnel/parking garage	High	High	High	Low	High	High	Total GNSS outage; inertial drift accumulation over time

**Table 5:** Comparative summary of multi-sensor fusion architectures (Kalman, particle filter, factor graph, deep BEV, transformer, SSM/Mamba, resilient MoE) across uncertainty handling, strengths, limitations, and best-fit applications.

Architecture	Fusion Level	Uncertainty Handling	Core Strengths	Limitations/Caveats
Kalman/EKF/UKF	Mid (state)/Late (tracks)	Explicit covariance propagation	Efficient; interpretable; real-time; integrates cleanly with control loops	Model-mismatch fragility; outlier sensitivity; Gaussian assumption
Particle Filter	Mid (state)	Sample-based posterior	Non-Gaussian and multi-modal belief support; handles strong nonlinearities	Computationally heavy; particle degeneracy; scales poorly with state dimension
Factor Graph/SLAM	Mid (constraints)	Probabilistic factor models	Flexible multi-constraint global consistency; handles asynchronous data	High optimization cost; robustification required for outlier factors
Deep BEV Fusion	Mid (features/repr.)	Learned, often implicit heads	Strong multi-modal perception; shared spatial representation	Data-hungry; OOD risk; strict calibration dependence; deployment cost
Transformer Fusion	Mid (features/queries)	Optional uncertainty heads	Soft cross-modal association; resilient to calibration drift	High architectural complexity; latency; heavy memory footprint
SSM/Mamba	Temporal/sequence	Implicit state uncertainty	Linear-time complexity; low latency; efficient decode for long sequences	Complex hyperparameter tuning; newer, limited automotive deployment evidence
Resilient MoE (MoME)	Late/decoding	Expert routing quality scores	Dynamic sensor failure recovery: state-of-the-art on nuScenes-R benchmark	Relies on accurate feature quality scoring; more complex training pipeline

**Table 6:** Sensor-centric safety requirements mapped to their technical architectural mechanisms across ISO 26262, ISO 21448 (SOTIF), ISO/PAS 8800, ANSI/UL 4600, and UNECE regulatory frameworks.

Safety Requirement Theme	Sensor-Centric Technical Implication	Typical Architectural Mechanism
Detect degraded sensing (ISO 26262 ASIL-B/D)	Identify partial hardware degradation before unsafe output is generated	Signal-level quality monitoring; cross-sensor innovation residuals; active contamination detection; self-test diagnostics
Bound localization integrity (ISO 26262/SOTIF)	Prevent biased global pose updates from entering planning and control	RAIM/ARAIM protection levels; robust fusion gating; HD map consistency checks; covariance inflation
AI model verification (ISO/PAS 8800)	Constrain non-deterministic neural network behavior within safe performance envelope	ISO/PAS 8800 AI lifecycle management; data bias testing; OOD performance benchmarking; epistemic UQ
Handle OOD scenarios (ISO 21448 SOTIF)	Avoid confident yet invalid inferences in novel environments or conditions	Energy-based OOD detection; epistemic uncertainty thresholds; runtime ODD monitoring; conformal prediction
Graceful degradation (ANSI/UL 4600)	Maintain minimal-risk condition when uncertainty or fault limits are exceeded	MoME-style expert routing; conservative planning fallback; safe-stop protocol; fault-tolerant trajectory
Evidence and traceability (UNECE GTR/UL 4600)	Demonstrate hazard controls and validation coverage to regulators across lifecycle	UL 4600 safety case artifacts; UNECE ISMR data logging; OpenSCENARIO scenario libraries; V&V coverage metrics

**Table 7:** Representative redundancy allocations across deployment targets, showing sensor diversity, algorithmic redundancy, compute isolation, and ODD breadth.

Redundancy Type	System Example	Primary Benefit	Key Architectural Caveat
Modal redundancy	Camera + radar + LiDAR fusion	Diverse physical failure modes; all-weather observability across optical, RF, and acoustic domains	Complex extrinsic calibration; cross-modal integrity checks required; common-mode algorithmic failure possible
Spatial/coverage redundancy	Corner + forward + rear radars; surround cameras	Occlusion reduction; full 360° coverage; eliminates blind spots at low speed	Still vulnerable to common RF clutter, multipath, or simultaneous physical contamination
Algorithmic diversity	EKF + factor graph + deep BEV running in parallel	Mathematical cross-checking; inconsistency-triggered alerts; reduces common-mode inference failure	High software complexity; extensive verification burden; increased compute and power requirements
Architectural isolation (Safety Island)	Dedicated lockstep microcontroller for monitoring and fallback	Enforces deterministic fallback even if primary AI accelerator stalls or violates timing	Requires mixed-criticality RTOS; strict power domain isolation; complex certification (ISO 26262 ASIL-D)
Temporal redundancy	Multi-frame integration; historical state smoothing	Buffers against transient sensor glitches; improves tracking continuity through brief outages	Adds latency; requires careful management of stale state; outdated measurements must be identified and excluded

**Table 8:** Representative autonomous driving architectural philosophies with sensor suites, robustness levers, primary risk concentrations, and deployment status [43,44,99,100,101,102].

Archetype	Sensor Suite (Verified Counts)	Key Robustness Lever	Primary Risk Concentration	Deployment Status
Waymo 6th-gen (LiDAR-centric multi-modal)	~13 cameras; 4 LiDAR; 6 imaging radars; audio	Modal redundancy; cross-modal consistency; HD-map priors	Adverse weather edge cases; calibration complexity; map staleness	Commercial robotaxi (Phoenix, SF, LA, Austin, Atlanta)
Tesla FSD (vision-centric)	8 cameras; optional forward radar (HW4)	End-to-end learning; fleet-scale data; OTA updates	Epistemic risk under OOD; no direct range sensing; perception failure modes	L2 supervised feature; large-scale deployment
Zoox (bidirectional robotaxi)	Camera + LiDAR + radar + LWIR (4 pods)	Uncorrelated LWIR modality; full 360° coverage; symmetric design	Platform non-standardization; limited public validation data	Driverless service (Las Vegas)
Cruise (hybrid multi-modal, historical)	~16 cameras; 5 LiDAR; ~21 radars	High redundancy; geofenced ODD; safety-case documentation	Governance/incident response; mode-transition safety	Program halted (Dec 2024)
Cooperative V2X-augmented	Onboard sensors + V2V/V2I (J2735, ETSI CPS)	Beyond line-of-sight awareness; infrastructure sensing	Latency; trust/verification; cybersecurity attack surface	Emerging deployments; regulatory transition to C-V2X

**Table 9:** The mapping between perception outputs, uncertainty representations, and corresponding planning and control actions is grounded in established risk-aware decision-making frameworks, including model predictive control (MPC), Responsibility-Sensitive Safety (RSS), and control barrier function (CBF)-based safety filters, which explicitly incorporate uncertainty into motion planning and constraint enforcement [39,108,109,110,111,114,116,117].

Perception/Fusion Output	Uncertainty Representation	Planning/Control Use	Failure Mode and Safety Action
Object detection and tracking	Covariance, multi-hypothesis tracks, and evidential scores	Collision avoidance, gap acceptance, lane-change feasibility	Underestimated covariance → unsafe interaction. Mitigate: chance-constrained margin inflation; reduced speed; conservative gap policy.
Free space/occupancy grid	Per-cell occupancy probability, confidence map	Drivable corridor generation, path feasibility	False free space under fog/backscatter → potential collision. Mitigate: cross-sensor validation; minimum-confidence floor; risk-inflated corridor.
Lane and HD-map alignment	Residual error, matching likelihood	Route following, lane-keeping constraints	Misalignment → lane departure. Mitigate: degraded lateral control mode; speed reduction; map-relative integrity check.
Ego pose (GNSS/IMU/odometry fusion)	State covariance, RAIM-style protection levels	Map-relative planning, intersection geometry	GNSS multipath bias → confidently wrong pose. Mitigate: down-weight GNSS; switch to feature/map localization; MRM if integrity is lost.
Object intent and trajectory prediction	Multi-modal trajectory distributions, mode probabilities	Interaction-aware planning, time-to-collision	Dropped mode/mode collapse → missed cut-in. Mitigate: maintain top-k modes with floor probability; expand reaction envelope.
Scene/weather classification	Categorical posterior, ODD-membership score	Speed governor, sensor weighting, ODD compliance	Misclassified condition → infeasible braking distance. Mitigate: conservative dynamic model; geofenced speed cap.
OOD/novelty score	Epistemic uncertainty, evidential mass on the unknown	Mode switching, policy gating	Missed OOD → overconfident maneuver. Mitigate: degraded mode entry; restrict ODD; trigger MRM with hysteresis.
Sensor health and integrity	Cross-sensor residuals, innovation gates, fault flags	Redundancy activation, decision gating, fallback arming	Undetected drift or contamination → silent miscalibration. Mitigate: fault isolation; switch to redundant modality; safe stop.

## Data Availability

No new data were created or analyzed in this study. Data sharing is not applicable to this article.
